# Transcriptional and epigenomic changes in response to polyethylene glycol-triggered osmotic stress in *Brassica napus* L.

**DOI:** 10.1093/jxb/eraf123

**Published:** 2025-04-03

**Authors:** Melvin Prasad, Prateek Shetty, Avik Kumar Pal, Gábor Rigó, Kamal Kant, Laura Zsigmond, István Nagy, Padubidri V Shivaprasad, László Szabados

**Affiliations:** Institute of Plant Biology, HUN-REN BRC, Szeged, Hungary; Institute of Plant Biology, HUN-REN BRC, Szeged, Hungary; National Centre for Biological Sciences, Tata Institute of Fundamental Research, Bangalore, India; Institute of Plant Biology, HUN-REN BRC, Szeged, Hungary; Institute of Plant Biology, HUN-REN BRC, Szeged, Hungary; Institute of Plant Biology, HUN-REN BRC, Szeged, Hungary; Seqomics Kft, Mórahalom, Hungary; National Centre for Biological Sciences, Tata Institute of Fundamental Research, Bangalore, India; Institute of Plant Biology, HUN-REN BRC, Szeged, Hungary; University Clermont Auvergne, France

**Keywords:** ChIP, DNA methylation, drought-responsive genes, histone methylation, H3K4me3, H3K27me3, *P5CS* genes, proline, rapeseed, transcriptome

## Abstract

Drought hinders growth, development, and productivity of higher plants. While the physiological and molecular background of plant responses to drought has been extensively studied, the role of post-translational modifications of histones or DNA methylation in response to dehydration remains largely elusive. In this study, we deciphered genome-wide changes in transcriptome and histone modifications in response to dehydration in rapeseed (*Brassica napus* L.). High-throughput transcript profiling (RNA-seq) and ChIP followed by sequencing (ChIP-seq) of polyethylene glycol (PEG)-treated rapeseed plants revealed genome-scale changes in transcription and histone methylation patterns, specifically in histone H3 lysine 4 trimethylation (H3K4me3) and histone H3 tri-methylated lysine 27 (H3K27me3) sites. We have identified gene sets with altered transcript profiles as well as histone methylation marks in response to osmotic stress. Several proline biosynthesis regulatory genes coding for Delta 1-Pyrroline-5-Carboxylate Synthetases (P5CS) displayed changes in H3K4me3 and/or H3K36me3 enrichment post-PEG treatment. Targeted bisulfite sequencing further identified stress-dependent gene body DNA methylation in one of the *BnP5CSA* gene copies that correlates with its stress-induced activation. By integrating physiological, transcriptional, and epigenomic data, our study contributes to a better understanding of the drought response control in crop plants.

## Teaser

We describe genome-wide transcript and epigenetic profiling of rapeseed in response to osmotic stress. A correlation was found between gene activation and H3K4me3 marks in several loci including *P5CSA* genes controlling proline accumulation.

## Introduction

Plants are continuously exposed to a changing environment, and extreme conditions such as drought, salinity, and high or low temperatures can have detrimental effects on their growth and development, triggering reduced photosynthesis, disrupted essential metabolic and physiological processes, and cytotoxicity (He *et al.*, 2018; Paes de Melo *et al.*, 2022; Zhang *et al.*, 2023). Drought limits crop productivity, posing a major threat to global food security (Iqbal *et al.*, 2020; Kapoor *et al.*, 2020). To thrive under these conditions, plants have evolved various mechanisms by adjusting their physiological, biochemical, and genetic parameters (Takahashi *et al.*, 2020). Epigenetic adjustments are reversible modifications affecting gene activation or silencing, and represent a significant regulatory aspect of plant adaptation to stress. The plant genome can undergo various epigenetic modifications, such as changes in DNA methylation patterns, histone modifications, and the production of small RNAs (Matzke and Mosher, 2014; Zhang *et al.*, 2018; Chang *et al.*, 2020; Miryeganeh, 2021).

DNA methylation controls gene expression, transposon silencing, and chromosome interactions. While DNA methylation in promoters typically represses gene expression, it can enhance transcription in gene bodies, where methylation predominantly occurs in the CG context. DNA methylation changes enable plants to adapt to stress conditions faster by facilitating the expression of stress-responsive genes (Zhang *et al.*, 2006; Liu *et al.*, 2010; Takuno and Gaut, 2013; Lei *et al.*, 2015; Williams *et al.*, 2015; Lang *et al.*, 2017; Zhang et al., 2018).

Histone modifications, such as methylation, acetylation, phosphorylation, and SUMOylation, can influence gene expression in response to abiotic stresses in plants (Liu *et al.*, 2012; Fang *et al.*, 2014; Kim *et al.*, 2015; Wang *et al.*, 2015; He et al., 2022; Sun *et al.*, 2022). These chemical modifications can alter chromatin compactness and gene accessibility, allowing the activation or repression of specific stress-responsive genes (Nunez-Vazquez *et al.*, 2022). Histone acetylation on lysine residues in H3 or H4 decreases their affinity for DNA, reduces chromatin compactness, increases accessibility to transcription factors, and subsequently tends to promote transcription (Shahbazian and Grunstein, 2007). Highly acetylated histones are often associated with active genes, while de-acetylated histones are more frequent in inactive genes. The effects of histone methylation are more complex, and depend on the amino acid residues involved. The methylation of Lys4 in H3 (H3K4me3) is an active mark, allowing a higher level of gene expression, while the methylation of Lys27 in H3 (H3K27me3) is considered a repressive mark, which appears frequently in repressed genes in heterochromatin (Nunez-Vazquez *et al.*, 2022; Sun *et al.*, 2022). Histone modifications are conserved and are mediated by similar enzymes in all eukaryotes: histone acetyltransferases (HATs) and histone deacetylases (HDACS) perform histone acetylation and deacetylation reactions, respectively, while histone methyltransferases (HMTs) aggregate and histone demethylases (HDMs) remove methyl groups. Activities of these enzymes are therefore important in determining histone modifications and can profoundly influence gene expression profiles in eukaryotic organisms (Pandey *et al.*, 2002; Pontvianne *et al.*, 2010; Nunez-Vazquez *et al.*, 2022). The regulation of gene expression through epigenetic modifications is vital for plant adaptability to stress, as it enables a dynamic and reversible response to environmental changes (Miryeganeh, 2021; Abdulraheem *et al.*, 2024). Reversible changes in H3K4me3 and H3K27me3 marks are often associated with changes in stress-dependent gene expression and were suggested to be essential components of adaptation to harmful conditions (Kim *et al.*, 2015; Nunez-Vazquez *et al.*, 2022). Furthermore, abiotic stress can influence the production and function of small RNAs, such as miRNAs and siRNAs, which target and regulate the expression of genes involved in stress (Matzke and Mosher, 2014). These small RNAs also mediate the silencing of transposable elements (TEs), thereby protecting the genome from stress-induced instability (Sunkar *et al.*, 2007; Kamthan *et al.*, 2015). Characterizing these epigenetic adjustments is essential for understanding responses to abiotic stress and revealing the molecular basis of plant adaptation to extreme environmental conditions (Singroha and Sharma, 2019; Ashapkin *et al.*, 2020).

A significant amount of our understanding of epigenetic regulation has been derived from studies on model organisms such as Arabidopsis. Knowledge of the epigenetic profiles of crops under stress can facilitate the development of novel strategies for creating stress-tolerant varieties through epigenetic engineering or targeted breeding programmes. Polyploidy-dependent changes in epigenetic regulation such as hypomethylation can promote responses to adverse environmental stimuli (Yuan *et al.*, 2020; L. Wang *et al.*, 2021). Advancements in high-throughput sequencing techniques have revolutionized the identification of epigenetic changes on a genomic level and facilitated our understanding of their impact on gene expression regulation (McCombie *et al.*, 2019). Information on stress-related epigenetic regulation of crops with large and polyploid genomes such as the allotetraploid rapeseed (*Brassica napus*) is, however, scarce. A comprehensive epigenome map of rapeseed has been published, providing genome-scale information on histone modifications, DNA methylation, and RNA polymerase II occupancy. Asymmetric histone modifications have been found between the rapeseed An and Cn subgenomes, as well as between various tissue types (Zhang *et al.*, 2021). Differences in epigenetic marks between rapeseed subgenomes have recently been reported which correlated with changes in transcript profiles (Hu *et al.*, 2023). Circadian oscillation of histone modifications in rapeseed have also been described, pointing to differential regulation of homologous gene pairs of the subgenomes (Xue *et al.*, 2023). Another study characterized the epigenetic modification of TEs in the evolution of the allopolyploid nature of rapeseed (Xiao *et al.*, 2022). While these studies have generated valuable datasets, revealing important information about the epigenome map, chromatin states, and regulatory elements on a genomic scale, epigenetic control of responses to environmental stresses such as drought in rapeseed is still unknown.

In this study, the widely cultivated rapeseed cultivar Westar (Klassen *et al.*, 1987) was subjected to osmotic stress using polyethylene glycol (PEG) treatment. Comprehensive transcriptome analyses identified drought-responsive differentially expressed genes (DEGs) involved in tolerance mechanisms. We performed H3K4me3 and H3K27me3 ChIP-seq assays on leaf tissue to characterize the genomic distribution of these marks in control and PEG-treated plants. This allowed us to link the enrichment or depletion of active and repressive histone modifications to their target genes. Our RNA-seq and ChIP-seq profiling revealed that several rapeseed *P5CSA* genes are activated by osmotic stress which displayed enriched H3K4me3 marks after PEG treatment. Besides histone modifications, rapid redistribution of DNA methylation marks upon osmotic stress in one of the *P5CSA* genes could be confirmed. This study represents the first transcriptomic and genome-wide H3K4me3 and H3K27me3 ChIP-seq analysis in rapeseed under osmotic stress, broadening our understanding of epigenetic marks in plant stress responses. Our findings provide valuable insights into the genetic and molecular mechanisms of stress response of an allotetraploid crop with a complex genome, and offer genetic resources for the improvement of high-yielding, drought-tolerant oilseed rapeseed varieties.

## Materials and methods

### Plant material and growth conditions

Plants of *Brassica napus* var. Westar (Klassen *et al.*, 1987) were grown on a hydrophonic system using Hoagland nutrient solution as described (Wang *et al.*, 2019). Plants were maintained and all treatments were performed in a growth chamber (Fytoscope SW, PSI, Czech Republic) under precisely controlled conditions. Illumination was provided with LED panels with a photon flux of 160 μmol photons m^–2^ s^–1^ and a short-day light cycle (8 h light at 22 °C/16 h darkness at 20 °C). Four-week-old plants were subjected to osmotic stress by replacing the standard Hoagland nutrient solution with solution supplemented by 20% PEG8000. Plants were allowed to recover from osmotic stress by replacing the PEG-containing solution with standard Hoagland solution. Leaf samples of PEG-treated and control plants were collected at various time points, specifically at 6 h, 24 h, 48 h, 3 d, 5 d, 7 d, 10 d, and 15 d, immediately frozen in liquid nitrogen, and stored until processing (the workflow is shown in Supplementary Fig. S1A). Experiments were repeated three times (biological replicates).

### Determination of proline content

Proline content was determined as described, with minor modifications (Kovács *et al.*, 2019). A 50 mg aliquot of leaf tissue was ground with liquid nitrogen, and 1 ml of 1% sulfosalicylic acid was added and mixed by vortex. The mixture was centrifuged at 12 200 *g* for 10 min at 4 °C, and 200 μl of the supernatant was mixed with 400 μl of 1.25% ninhydrin reagent (ninhydrin dissolved in 80% acetic acid). The samples were incubated in a dry block heater bath at 95 °C for 30 min, and immediately placed on ice for several minutes. Proline content was determined by measuring the absorbance of the reaction product at 520 nm using a Thermo Scientific, Multiscan Go Microplate Spectrophotometer. To draw the standard curve of proline, concentrations of 0.5, 0.25, 0.125, 0.025, 0.03125, and 0 mM proline were used as reference. The content of proline was measured with five technical replicates.

### Determination of malondialdehyde

Lipid peroxidation rates were measured by the thiobarbituric acid-reactive substances (TBARS) assay as described (Heath and Packer, 1968). A 100 mg aliquot of leaf tissue was homogenized in 1 ml of 0.1% trichloroacetic acid (TCA) containing 0.4% butylhydroxytoluene, centrifuged at 12200 g for 20 min. Then 1 ml of 20% TCA containing 0.5% thiobarbituric acid (TBA) was added to 250 μl of the supernatant, mixed, and incubated at 96 °C for 30 min. Absorbance was measured at 532 nm using a Multiskan GO microplate reader (Thermo Fisher Scientific) and calculated by subtracting the non-specific absorbance measured at 600 nm. Malondialdehyde (MDA) concentration was calculated using the extinction coefficient ε532=155 mM^−1^ cm^−1^. Three biological replicates were made.

### Determination of photosynthetic parameters

Chlorophyll fluorescence measurements on light-adapted leaves were carried out directly in the growth chamber using a portable MultispeQ v1 device (PhotosynQ) controlled by the PhotosynQ platform software (Kuhlgert *et al.*, 2016), using a red actinic light (200 μmol photons m^−2^ s^−1^) and a 500 ms saturation pulse (3000 μmol photons m^−2^ s^−1^).

### RNA extraction and quantitative reverse transcription–PCR analysis

Total RNA was isolated using a GeneJET RNA Purification Kit (Thermo Scientific) following the manufacturer’s protocol. A 1 μg aliquot of DNase-treated RNA was used for cDNA synthesis using the High-Capacity cDNA Reverse Transcription Kit (Applied Biosystems). Quantitative reverse transcription–PCR (RT–qPCR) was performed on 3 μl of 20× diluted cDNA templates using 5× HOT FIREPol EvaGreen qPCR Mix Plus (Solis Biodyne) in a final volume of 10 μl, and a Bio-Rad CFX96 Touch Deep Well Real-Time PCR System. Mean values of actin and glyceraldehyde phosphate dehydrogenase (GAPDH) Ct were used as internal reference. Normalized relative transcript levels were determined using the 2^–ΔΔCt^ method (Livak and Schmittgen, 2001). Experiments were repeated with three biological replicates. Oligonucleotides used in this study are listed in Supplementary Table S1.

### RNA-seq analysis

Total RNA was isolated using a GeneJET RNA Purification Kit (Thermo Scientific) following the manufacturer’s protocol. Approximately 3 μg of total RNA from each sample was subjected to the RiboMinus Eukaryote Kit (Qiagen, Hilden, Germany) to remove rRNA prior to the construction of the RNA-seq libraries. RNA-seq libraries were prepared using an RNA-seq Library Prep Kit for Illumina (New England Biolabs). The libraries were sequenced on an Illumina HiSeq 4000 platform using a paired-end 150 bp read module. The raw sequencing reads were filtered and trimmed using the default parameters of FastQC (version 0.11.5). The filtered clean data were then assembled and compared with the reference genome of *B. napus* (http://cbi.hzau.edu.cn/rape/download_ext/westar.genome.fa) using the hisat2 tool (Version 2.2.1, http://ccb.jhu.edu/software/hisat2) with the default parameters. The FPKM (fragments per kilobase of transcript per million mapped reads) was calculated using the Cufflinks tool (version 2.2.1, http://cole-trapnell-lab.github.io/cufflinks/), and DEGs were identified using DESeq2 (https://bioconductor.org/packages/devel/bioc/vignettes/Glimma/inst/doc/DESeq2.html with the criteria of *q*-value (adjusted *P*-value, Benjamini–Hochberg method) <0.05 and |log_2_ (fold change, FC)| ≥2. Plots were generated with the R package ggplot2. Correlation analysis was performed by R (V3.3.2; https://www.r-project.org/). The correlation of two parallel experiments provides the evaluation of the reliability of experimental results as well as operational stability. The correlation coefficient between three replicas was calculated to evaluate repeatability between samples. Principal component analysis (PCA) was performed with the R package gmodels (https://www.rproject.org/). Functions of the DEGs were investigated with Gene Ontology (GO) pathway analysis using topGO (version 4.4) and ClusterProfiler (version 4.12.2) (T. Wu *et al.*, 2021), respectively. Significant GO terms and Kyoto Encyclopedia of Genes and Genomes (KEGG) pathways were identified with the criterion of *q*-value <0.05. Sequence data were deposited in the National Center for Biotechnology Information (NCBI) SRA database (accession number: PRJNA1144338). Venn diagrams were generated using the Bioinformatics and Evolutionary Genomics web tool available at https://bioinformatics.psb.ugent.be/webtools/Venn/.

### ChIP-seq and ChIP-qPCR

ChIP assays were performed as previously described by Song *et al.* (2016) with minor modifications. In brief, 2–3 g of 30-day-old plant leaves (Mock and PEG treated) were cross-linked with 1% formaldehyde for 20 min under vacuum, quenched with freshly prepared 2 M glycine, and ground into fine powder in liquid nitrogen. Chromatin was isolated and sheared into 200–500 bp DNA fragments by sonication. The sonicated chromatin was immunoprecipitated with one of the following antibodies (5 μg): anti-trimethyl-histone H3 (Lys4) (Merck, 07-473), anti-histone H3K27me3 (active motif, 39155), anti-histone H3 (di-methyl K9) (Abcam, ab1220), anti-histone H3 (tri-methyl K36) (Abcam ab9050), anti-histone H4 (acetyl K5) (Abcam, ab51997), or anti-Myc tag antibody (used as the IgG negative control, Abcam, ab9E11), and with 25 μl of Dynabeads Protein G (Invitrogen, 10003D) for 12 h at 4 °C with rotation. The precipitated chromatin DNA was then purified by phenol–chloroform–isoamyl alcohol extraction and recovered by ethanol precipitation. The ChIP DNA was prepared for sequencing or qPCR. Two biological replicates were used for ChIP-seq, and three biological replicates were used for ChIP–qPCR. ChIP-IgG was used for normalizing the values. The primers used for ChIP qPCR are listed in Supplementary Table S2. Actin (BnaC09T0320800WE) was used as a negative control.

### ChIP-seq data analysis

At least 10 ng of ChIP DNA was used for library preparation. ChIP sequencing libraries were constructed using the NEBNext Ultra II DNA Library Prep Kit (New England Biolabs Inc., E7103) following the manufacturer’s instructions. Constructed libraries were sequenced using Illumina NovaSeq 6000, and paired-end reads were obtained using Geninus Bowtie2 software (https://www.kr-geninus.com) (Langmead and Salzberg, 2012) and were used to align the sequencing reads of ChIP-seq to the *Brassica_napus* reference genome (http://cbi.hzau.edu.cn/rape/download_ext/westar.genome.fa) utilizing default parameters. The peak in different conditions and differentially changed peaks were called by MACS software (Zhang *et al.*, 2008). The nomodel parameter was set, and the d-value parameter was set at 200. The resulting wiggle files, which represent counts of ChIP-seq reads across the reference genome, were normalized for sequencing depth by dividing the read counts in each bin by the millions of mapped reads in each sample and were visualized in the IGV genome browser. Diffbind was used to compute differentially bound sites between PEG-treated and control conditions from multiple ChIP-seq experiments using affinity (quantitative) data. For the analysis of histone modification profiles between the 24 h PEG-treated samples and the Mock-treated samples, the Galaxy platform was employed (https://usegalaxy.eu). Sequence data were deposited in the National Center for Biotechnology Information (NCBI) SRA database (accession number: PRJNA1144218).

### Targeted DNA methylation

Primers for the targeted DNA methylation were designed using the Bisulfite Primer Seeker tool (https://zymoresearch.eu). DNA from the rapeseed was isolated using the DNA extraction kit Nucleon PhytoPure (Merck). Bisulfite conversion of DNA was carried using the EZ DNA Methylation-Gold Kit (Zymoresearch, D5005). Bisulfite-converted DNA was used as a template for the targeted DNA amplification. PCR fragments were purified and used for sequencing. Primers for targeted bisulfite sequencing analysis are listed in Supplementary Table S3. Adapters were removed from the deep sequenced PCR products by the cutadapt tool (Martin, 2014). For alignment of the reads, the target sequence was converted by Bismark aligner. Adapter trimmed sequences (90–100 bp) were aligned to the target site using the Bismark aligner tool with default parameters (Krueger and Andrews, 2011). The DNA methylation status of the targeted site was extracted and coverage reports were generated using the Bismark aligner tool. The obtained results were analysed using the methylation package ViewBS (Huang *et al.*, 2018). Sequence data were deposited in the National Center for Biotechnology Information (NCBI) SRA database (accession number: PRJNA1144360).

### Statistical analyses

Statistical analysis was performed using two-way ANOVA followed by the post-hoc Tukey HSD test (*P*<0.05) using the Graphpad prism software (version 8). The data presented in graphs represent the mean value ±SE of three independent experiments.

## Results

### Consequences of PEG-induced osmotic stress on rapeseed

**Fig. 1.**
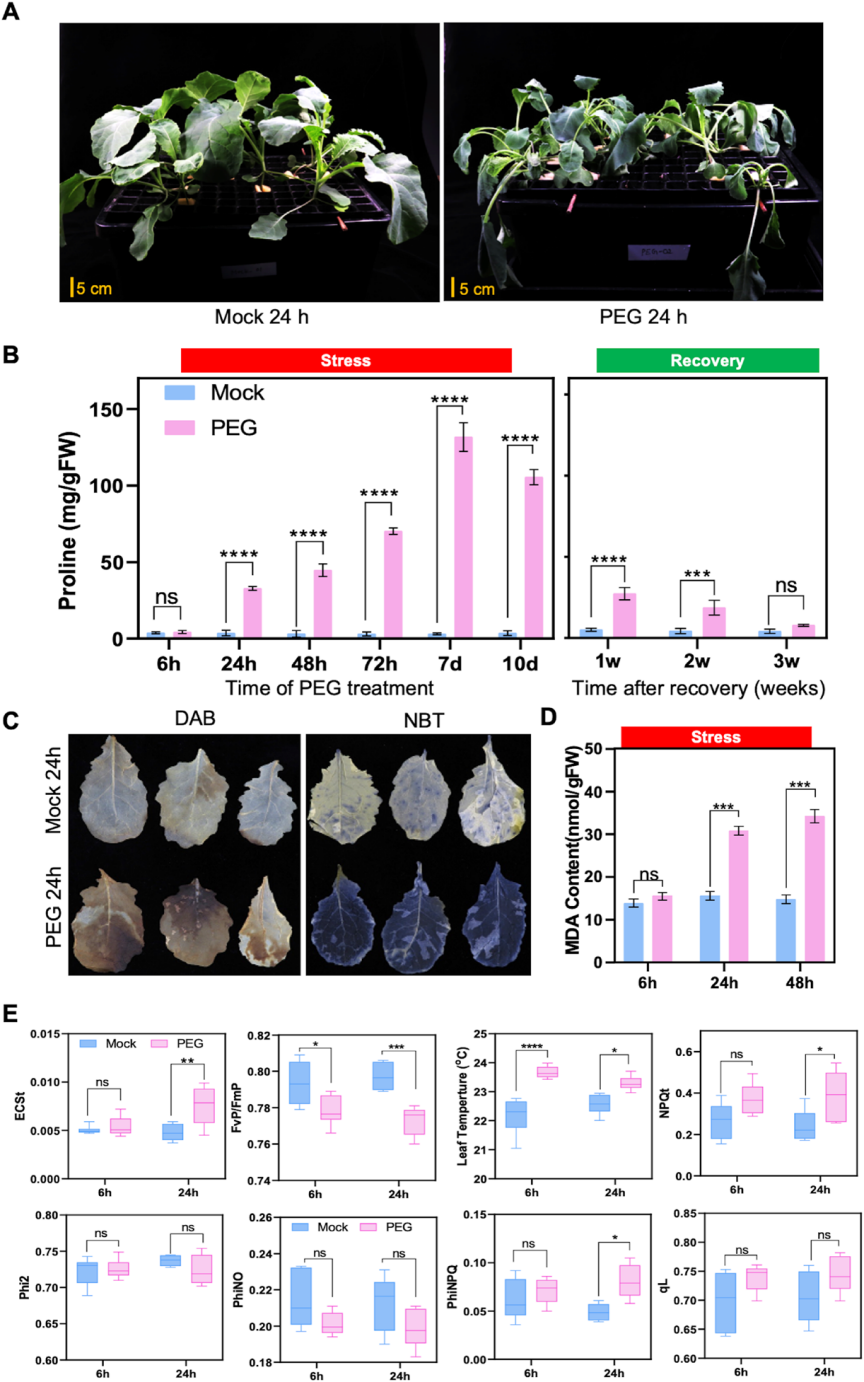
Effect of osmotic stress on rapeseed plants. (A) Morphological changes in rapeseed plants after 24 h of 20% PEG treatment in hydroponic culture. (B) Proline levels in leaves of rapeseed plants during osmotic stress and after recovery. (C) Accumulation of ROS in stressed (PEG) and control (Mock) rapeseed plants. Histochemical detection of hydrogen peroxide (3,3-diaminobenzidine, DAB) and superoxide levels (nitroblue tetrazolium, NBT) staining in rapeseed leaves. (D) Lipid peroxidation measured as malondialdehyde (MDA) accumulation in leaves of rapeseed plants. (E) Effect of osmotic stress on photosynthetic parameters of PSII in rapeseed plants: energy-dependent quenching (ECSt), maximum quantum efficiency of PSII (FvP/FmP), quantum yield of non-regulated energy dissipation (PhiNO), quantum yield of PSII photochemistry (Phi2), leaf temperature (^o^C), non-photochemical quenching (NPQ), photochemical quenching coefficient (qL), and quantum yield of regulated energy dissipation (PhiNPQ). Bars represent the mean ±SE of three independent experiments. Statistical analysis was performed using a two-way ANOVA followed by Tukey’s multiple comparison test. ‘ns’ indicate no significant difference, while *P<0.05, **P<0.01, ***P<0.001, and ****P<0.0001 indicate statistically significant differences.

Water deprivation during drought generates osmotic stress in plants which can be simulated by PEG treatment (Wang *et al.*, 2019). To subject rapeseed to osmotic stress, a hydroponic system was employed with 20% PEG8000 as osmotic agent (Supplementary Fig. S1A). Rapeseed plants exhibited immediate signs of water loss, which manifested as wilting within several hours of PEG treatment (Fig. 1A).

Proline accumulation in higher plants is a characteristic response to drought or high soil salinity, and is often considered as a marker for altered physiological homeostasis (Alvarez *et al.*, 2022). PEG-treated plants displayed significant proline accumulation, where leaves had the highest level of proline, while stems and roots had negligible proline accumulation (Supplementary Fig. S1B). Subsequent assays were therefore performed with leaves. When time-dependent proline accumulation was tested, a gradual increase in proline content was observed from the 6 h time point through to the 7 d interval, then proline levels gradually declined. When PEG was replaced with standard nutrient solution, proline levels were reduced to control in 3 weeks (Fig. 1B).

Accumulation of reactive oxygen species (ROS) in stressed plants is known to generate oxidative damage. Hydrogen peroxide and superoxide levels were analysed in PEG-treated rapeseed with DAB (3,3'-diaminobenzidine) and NBT (nitroblue tetrazolium) histochemical reactions, respectively. Both assays generated intensive colour reactions in PEG-treated rapeseed, but not in controls (Fig. 1C). Lipid peroxidation is detected through accumulation of MDA, an oxidative stress marker. High MDA accumulation indicated extensive oxidative damage in the PEG-treated plants but not in the control (Fig. 1D).

To further characterize physiological responses, photosynthetic parameters were compared in PEG-treated and control plants. There was a notable decrease in maximum quantum efficiency of PSII in the light-adapted state (FvP/*F*mP) and quantum yield of non-regulated energy dissipation (PhiNO) in the stressed plants. The electron transport rate through PSII (ECSt), non-photochemical quenching (NPQ), and quantum yield of regulated energy dissipation (PhiNPQ), as well as leaf temperatures, were higher in PEG-treated plants. No significant differences were detected in photochemical quenching coefficient (qL) and quantum yield of PSII photochemistry (Phi2) (Fig. 1E). These findings suggest that PEG generates specific changes in the photosynthetic performance of rapeseed.

**Fig. 2.**
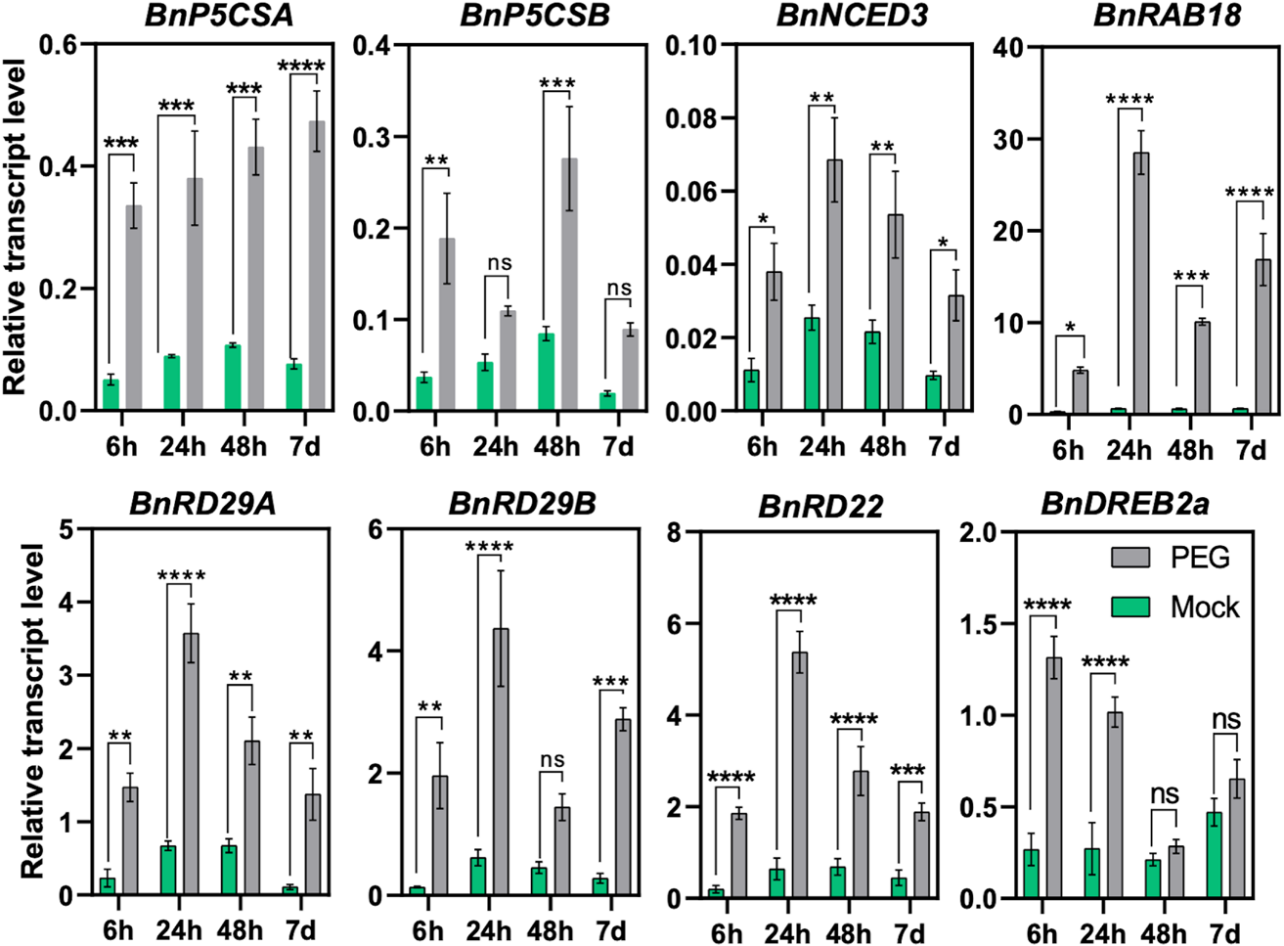
Response of stress marker genes to PEG-induced osmotic stress. Transcript levels of selected genes were determined by RT–qPCR in PEG-treated and control rapeseed plants. Data represent the fold induction of each gene by osmotic stress relative to the *BnACT* transcript levels as a reference. Bars represent the mean ±SE of three independent experiments. Statistical analysis was performed as indicated in Fig. 1.

Proline levels are determined by the activities of biosynthetic and catabolic pathways, controlled by Delta-1-pyrroline carboxylate-5-synthase (P5CS), and by proline dehydrogenase (PDH) enzymes, respectively (Supplementary Fig. S2) (Szabados and Savouré, 2010; Mattioli *et al.*, 2022). In Arabidopsis, P5CS is encoded by two genes, *P5CS1* and *P5CS2*, with remarkable differences in their expression regulation (Savouré *et al.*, 1997; Strizhov *et al.*, 1997; Kovács *et al.*, 2019). In contrast to Arabidopsis, rapeseed possesses 10 *P5CS* genes, which belong to the *P5CSA* and *P5CSB* subgroups. Transcript levels of the A and B types of rapeseed *P5CS* has been determined by RT–qPCR, although this method cannot distinguish between individual genes due to their high sequence identities. Both *BnP5CSA* and *BnP5CSB* gene groups were rapidly activated in PEG-treated plants, with their transcript levels remaining elevated throughout the high osmotic conditions (Fig. 2). In Arabidopsis, stress-dependent *P5CS1* induction relies on both ABA-dependent and independent signals (Savouré *et al.*, 1997). To investigate whether similar regulatory mechanisms exist in rapeseed, the expression of selected abscisic acid (ABA)- and stress-responsive genes was monitored in PEG-treated and control plants.

In Arabidopsis, ABA biosynthesis is regulated by the stress-responsive *NCED3* (Finkelstein, 2013). Transcript levels of *BnNCED3* were enhanced by PEG, suggesting activation of ABA biosynthesis and signalling in rapeseed. Arabidopsis genes *RD29A*, *RD29B*, *RD22*, and *RAB18* are well-characterized drought- and ABA-induced genes (Takahashi *et al.*, 2018). In rapeseed, *BnRD29A*, *BnRD29B*, *BnRD22*, and *BnRAB18* genes were also induced by PEG (Fig. 2). Arabidopsis *DREB2* is induced by water deprivation independently of ABA (Liu *et al.*, 1998). Rapeseed *BnDREB2A* was induced by PEG treatment, suggesting an active ABA-independent regulation under high osmotic conditions (Fig. 2). These transcript data provide compelling evidence that the PEG treatment generates substantial changes in gene expression patterns and is suitable for conducting transcriptomic and epigenetic investigations in rapeseed.

### Genome-wide transcript analysis of rapeseed

**Fig. 3.**
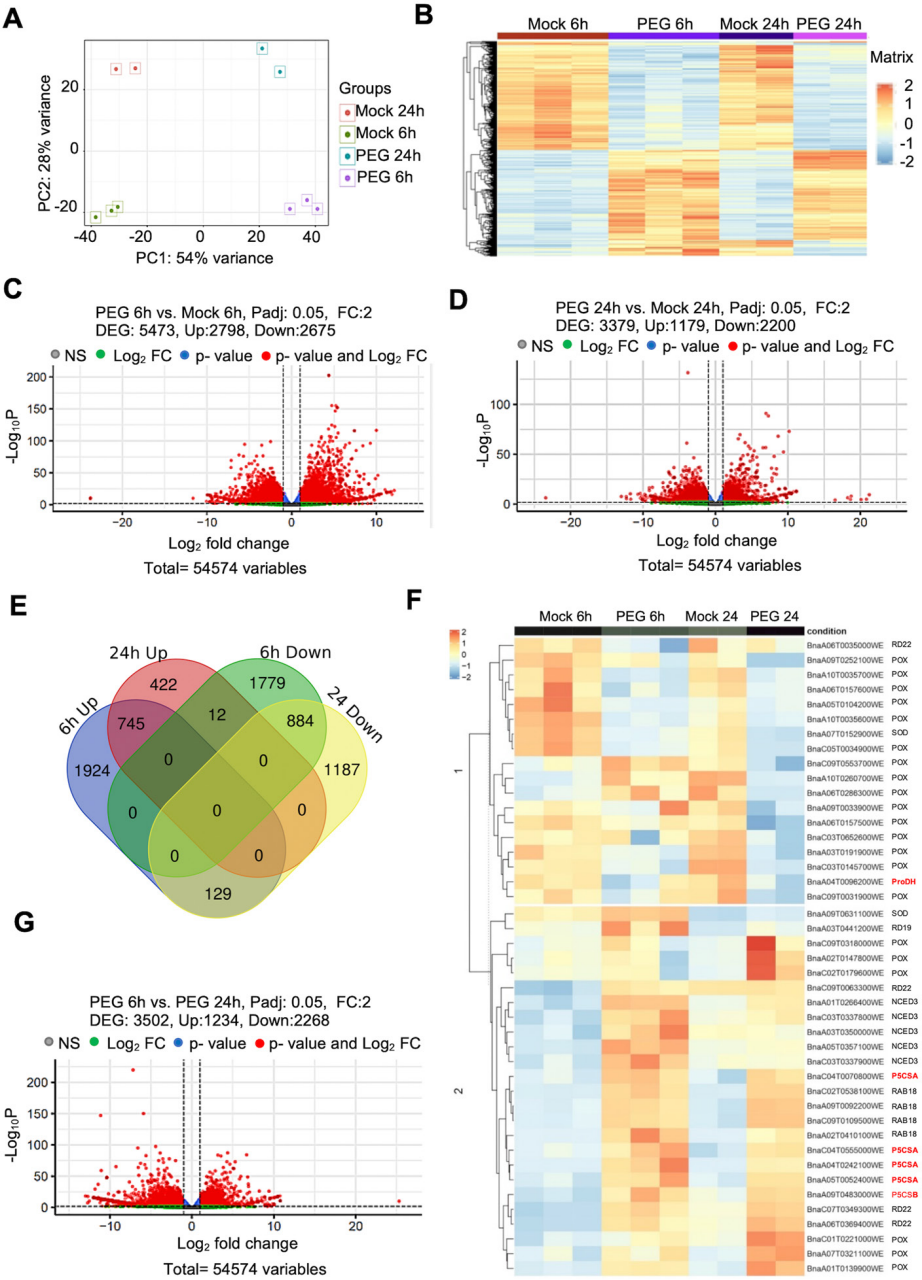
RNA-seq analysis of rapeseed in response to osmotic stress. (A) Principal component analysis (PCA) of transcript datasets obtained from Mock and PEG-treated plants. (B) Heatmap representation of gene expression, calculated as the average reads per kilobase of transcript per million reads mapped (RPKM) value from the biological replicates. (C, D) Volcano plot analysis of differentially expressed genes (DEGs) in Mock and PEG-treated samples after 6 h (C) and 24 h (D) of PEG treatment. (E) Venn diagram of DEGs illustrating the overlap and unique sets of genes regulated at 6 h and 24 h of PEG treatments. (F) Expression heatmap of rapeseed stress-responsive genes at 6 h and 24 h after PEG treatment. (G) Volcano plot comparison of DEGs between 6 h and 24 h in Mock and PEG-treated samples (*P*adj <0.05, log_2_ FC >2).

To obtain information on genome-wide expression changes of rapeseed during osmotic stress, RNA-seq analysis was performed on 4-week-old plants subjected to 6 h and 24 h PEG treatments and their respective controls. The RNA-seq quality control reports are summarized in Supplementary Table S4. PCA of the RNA-seq data demonstrated that replicates cluster together and were clearly separated from their respective treatment groups, with the exception of two samples which were different from their respective groups (one Mock_24 h and one PEG 24 h sample) (Supplementary Fig. S3A). Initially, bioinformatic analysis was performed, including outliers. This analysis identified a total of 2720 genes that were differentially expressed in response to 20% PEG treatment at the 24 h time point. Of these, 1028 genes were up-regulated, while 1692 were down-regulated (Supplementary Fig. S3C; Supplementary Dataset S1). A total of 453 DEGs were unique to the analysis with two replicates, while 2267 DEGs (67%) were common between the two- and three-replicate analyses (Supplementary Dataset S2). To minimize noise in both RNA-seq and downstream ChIP analyses, we decided to exclude replicates showing significant variation, resulting in less variation and more coherent PCA results (Fig. 3A). Genes with differential expression patterns could be clustered together as displayed by a heatmap (Fig. 3B). In 6 h PEG-treated samples, our analysis identified 5473 DEGs, employing a statistical significance threshold of *P*adj 0.05 and an FC cut-off of 2.0. In response to PEG treatment, 2798 genes were up-regulated, while 2675 genes were down-regulated (Fig. 3C; Supplementary Fig. S3B; Supplementary Dataset S3). Transcriptomic analysis of the 24 h samples identified 3379 DEGs, with 1179 genes being up-regulated and 2200 genes down-regulated (Fig. 3D; Supplementary Fig. S3B, D; Supplementary Dataset S4). We compared the RNA-seq data generated with and without the inclusion of outliers. Approximately 67% of both up-regulated and down-regulated genes overlapped between the two- and three-replicate analyses (Supplementary Datasets S1, S2). To further illustrate this comparison, a Venn diagram was generated, highlighting the overlapping up-regulated and down-regulated genes between the two analyses (Supplementary Fig. S3E). While three biological replicates are usually employed in transcriptomic analysis, several previous studies have reported valid results with two replicates (Zhu *et al.*, 2018; T.Y. Wu et al., 2021). In subsequent studies, we used two replicates of the 24 h samples, with more coherent data.

A comparative analysis between the 6 h and 24 h samples revealed that 745 genes were up-regulated at both time points in response to PEG, while 884 genes were down-regulated. Intriguingly, a subset of 129 genes exhibited up-regulation after 6 h PEG treatment, which became down-regulated after 24 h of stress (Fig. 3E). Twelve genes were down-regulated at the 6 h PEG treatment, which became up-regulated after 24 h of stress (Fig. 3E). A comprehensive list of the genes displaying differential expression at the 6 h and 24 h PEG treatments can be found in Supplementary Datasets S3–S10.

GO analysis revealed that 6 h PEG treatment induced genes associated with responses to various stresses and stimuli, autophagy, and defences, while 24 h PEG lead to up-regulation of genes related to stress responses, lipid transport, and developmental processes (Supplementary Figs S4A, S5A). Genes linked to photosynthesis, peptide biosynthesis, and various metabolic pathways (sugar, lipids) were down-regulated by 6 h PEG treatment, while genes implicated in transport processes, and lipid and fatty acid metabolism were inhibited after 24 h of PEG (Supplementary Figs S4B, S5B).

In order to obtain information on regulatory genes, we assembled a list of defence-related transcription factors and proteins implicated in chromatin modifications, based on their sequence similarity to characterized Arabidopsis factors which are implicated in control of stress responses and/or determining chromatin structure. A substantial portion of transcripts from the *MYC* and *ABF*/*ABI* gene families displayed up-regulation at both 6 h and 24 h of PEG treatment. In contrast, most *WRKY* transcripts exhibited down-regulation (Supplementary Fig. S6). Among the genes implicated in chromatin regulation, transcript levels of several *HDAC*, *HAC1*, *DRM2*, *JMJ*, *SUVH6*, *ATX*, and *SUVR* genes showed higher expression, whereas *CLASSY1-like*, *MET1*, *DME*, *CMT2*, *HAC1*, and *HDAC1* genes displayed reduced expression under PEG stress (Supplementary Fig. S7).

The expression patterns of a subset of drought-responsive defence-related genes were analysed at 6 h and 24 h under drought stress. A heatmap presented the expression patterns of 43 DEGs. Most transcripts for *BnP5CSA*, *BnNCED3*, *BnRD22*, *BnRD19*, *BnRAB18*, and *BnSOD* were induced in response to PEG, while *POX* and *ProDH* were down-regulated (Fig. 3F).

We compared the gene sets differentially misregulated in 6 h and 24 h PEG-treated plants. We identified 3502 DEGs, with 1234 and 2268 genes displaying up- and down-regulation after 24 h of PEG when compared with 6 h treatments, respectively (Fig. 3G). These data revealed substantial changes in gene expression regulation of rapeseed plants exposed to short- and long-term osmotic stress.

### Epigenetic profiling of H3K4me3 and H3K27me3 marks

**Fig. 4.**
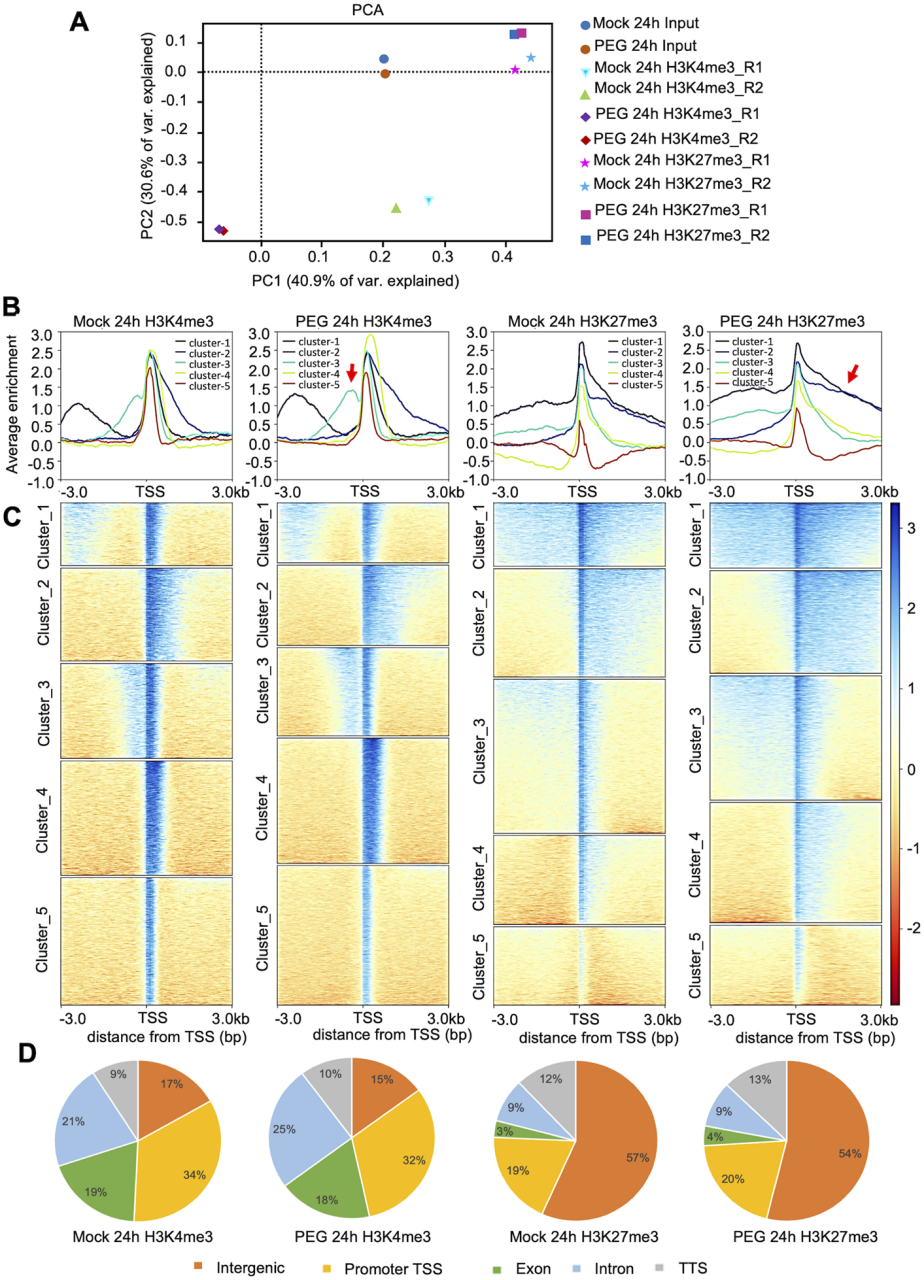
ChIP-seq analysis of rapeseed in response to PEG treatment. (A) PCA of Mock and PEG-treated datasets. (B) Average plot of ChIP-seq signals for H3K4me3 and H3K27me3 in Mock and PEG-treated samples. Meta-gene profiles were generated using the normalized sequencing density of H3K4me3 and H3K27me3. Stress-dependent change in the distribution of histone marks across the regions are indicated by arrows. (D) Heatmap of enriched ChIP-seq signals for H3K4me3 and H3K27me3 in five clusters. (E) Genome-wide distribution of H3K4me3 and H3K27me3 in different genomic regions.

Histone modifications play pivotal roles in the regulation of gene expression governing various aspects of growth, development, and defence mechanisms. To identify genome-wide epigenetic changes, ChIP-seq analysis was performed on leaf samples derived from plants subjected to 24 h PEG treatment and their respective controls. Comprehensive genome-wide profiling of two key histone marks was performed, identifying histone H3K4me3 and H3K27me3, associated with gene activation and gene repression, respectively. The ChIP-seq data encompassed a total of 10 libraries, comprising eight for immunoprecipitated DNA and two for input DNA. These libraries collectively yielded ~212 million paired-end reads, in the range of 15–21 million reads per sample (Supplementary Table S5). A total of 76% of the reads could be successfully mapped to the rapeseed reference genome (BnPIR: http://cbi.hzau.edu.cn/bnapus). Comparing the histone modification profiles of the PEG-treated and control samples, distinct variations in the principal components were revealed, differentiating between the two histone modifications (Fig. 4A). The replicates displayed a consistent signal for H3K4me3 and H3K27me3, indicating robust experimental reproducibility. The pairwise correlation among the demultiplexed samples exhibited high values, ranging from 0.71 to 1.0 (Supplementary Fig. S8). Comprehensive assessment of genome coverage and ChIP-seq enrichment was made employing fingerprint plot analysis. In the context of ChIP enrichment, the rightward deflection of the trace signifies the degree of enrichment observed. In this regard, all datasets exhibited ChIP enrichment when compared with the input (Supplementary Fig. S9A, B; Supplementary Datasets S11, S12).

Our plot profile analysis revealed distinctive patterns in the distribution of H3K4me3 and H3K27me3 marks on the rapeseed genome. Using a k-means clustering assay with H3K4me3- and H3K27me3-associated genes, we identified five clusters with distinct ChIP-seq read distributions (Fig. 4B, C). Clusters 1 and 3 exhibited broad H3K4me3 enrichment in both promoter and transcription start site (TSS) regions, while Cluster 2 showed H3K4me3 distribution in the gene body as well as in the TSSs. Clusters 4 and 5 displayed H3K4me3 enrichment at the TSS site only. For H3K27me3 distribution, Cluster 1 genes had marks in the promoter, TSS, and gene body regions, whereas Clusters 2 and 4 showed H3K27me3 marks in the gene body. Cluster 3 had H3K27me3 enrichment in the promoter and TSS regions, and Cluster 5 restricted H3K27me3 marks to the TSS. Comparing PEG-treated samples with controls, we observed subtle alterations in the plot profile trends for H3K4me3 marks. A slight increase in H3K4me3 marks was observed for Cluster 3 just before the TSS in the PEG-treated samples. Moreover, PEG induced a broader distribution and heightened enrichment of H3K27me3 marks compared with the control (Fig. 4B, C).

**Fig. 5.**
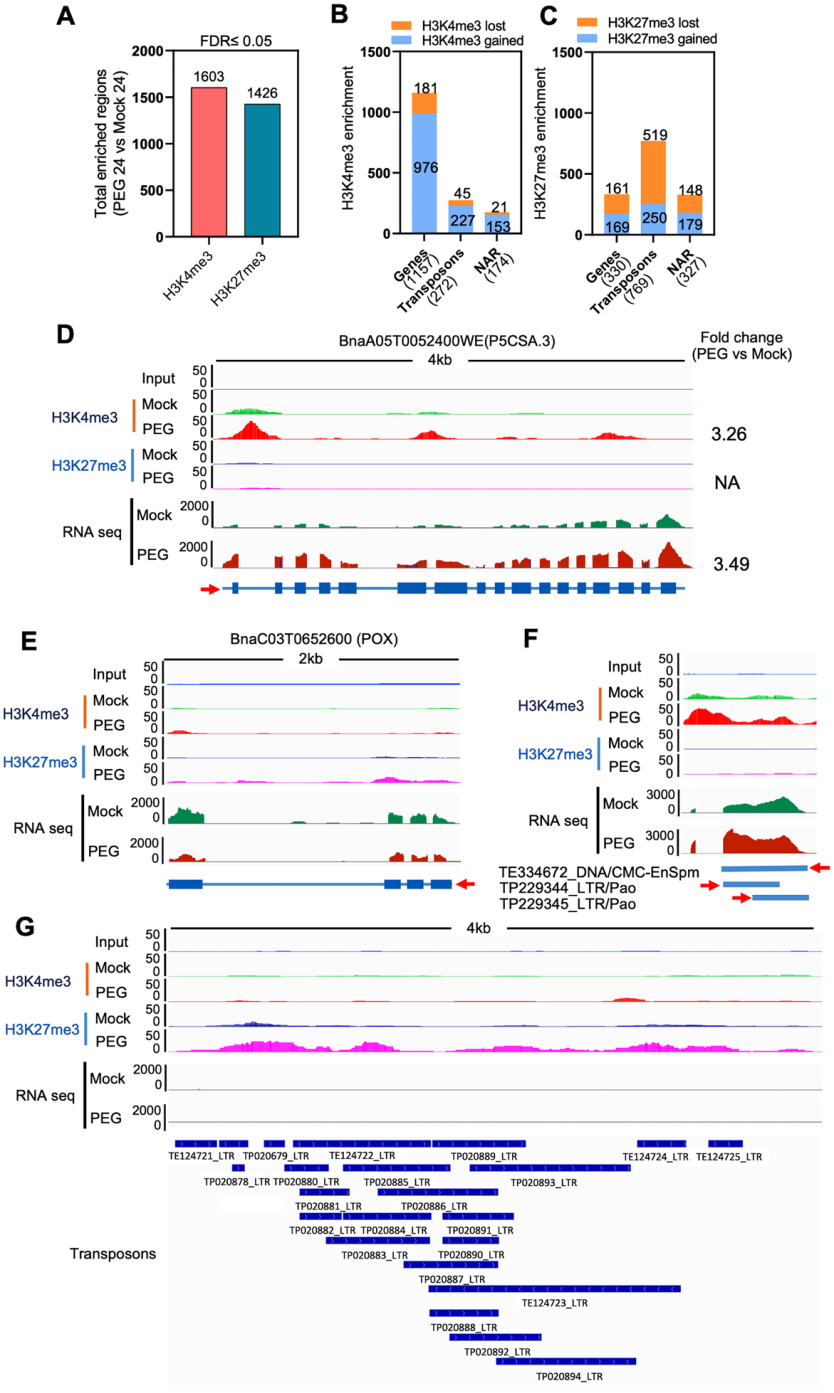
ChIP-seq for active histone marks H3K4me3 and H3K27me3. (A) Total enriched regions for H3K4me3 and H3K27me3 marks in PEG-treated samples compared with control (Mock). (B, C) Genomic distribution of H3K4me3 (B) and H3K27me3 (C) marks, showing the identified gained and lost sites in rapeseed under PEG treatment, in association with genes, transposons, and non-annotated regions (NAR). (D–F) ChIP-seq and RNA-seq tracks for the drought-responsive *BnP5CSA.3* (D), *POX* genes (E), and a region encoding TEs (F). The *y*-axis represents normalized read counts of H3K4me3, H3K27me3, and transcript reads (RPKM) within the locus (*x*-axis). The arrow indicates the direction of transcription. (G) ChIP-seq tracks of genomic regions enriched with H3K27me3 that encode various TEs. The *y*-axis represents normalized read counts of H3K27me3.

To visualize the distribution of marks across the genome, five annotated subregions were defined: promoters and TSSs, exons, introns, downstream TTSs, and distal intergenic regions. H3K4me3 and H3K27me3 distributions between these regions were different (Fig. 4D). PEG-treated samples showed a 1% reduction in H3K4me3 marks in intergenic and exon regions, and 2% in promoter–TSS regions. There was a 4% increase in intronic regions and a 1% increase in TTS regions for H3K4me3 marks. Intergenic regions had a 3% decrease, while promoter and TTS regions showed a 1% increase of H3K27me3 marks in PEG-treated samples. H3K27me3 marks were notably associated with transposon regions, with 250 gaining and 519 losing marks, while 330 genes displayed altered binding patterns (169 gaining and 161 losing marks), and 327 non-annotated regions showed H3K27me3 peaks (Fig. 5C).

Genomic distribution patterns of H3K4me3 and H3K27me3 marks were tested by differential binding (Diffbind) analysis on mapped reads, and revealed 36 998 enriched regions associated with H3K4me3 and 37 541 with H3K27me3. Applying stringent criteria (FDR ≤0.05 and log2FC ≥1) to normalize control H3K4me3-enriched regions, we identified 1603 regions with differential H3K4me3 binding (Fig. 5A; Supplementary Fig. S9C, D) and 1426 regions with differential H3K27me3 binding (Fig. 5A; Supplementary Fig. S9E, F). Enriched H3K4me3 regions encompassed 1157 genes, with 976 gaining and 181 losing binding, alongside 272 transposons (227 gaining and 45 losing binding), and 174 non-annotated regions (153 gaining and 21 losing binding) (Fig. 5B). Representative loci illustrated the distribution of H3K4me3 and H3K27me3 marks on mRNA transcripts of *BnP5CSA.3* and *BnPOX* genes (Fig. 5D, E) and TEs (Fig. 5F, G).

We analysed the distribution of H3K4me3 and H3K27me3 marks across TE regions under PEG stress. Enrichment of H3K4me3 and H3K27me3 was predominantly observed within retrotransposons, particularly those of the LTR-Copia, LTR-Gypsy, and LINE families, whereas such marks were less pronounced in DNA transposon regions. Our results revealed that the level of enrichment is different for these marks (Supplementary Figs S10, S11).

### Histone modifications associated with gene expression changes

**Fig. 6.**
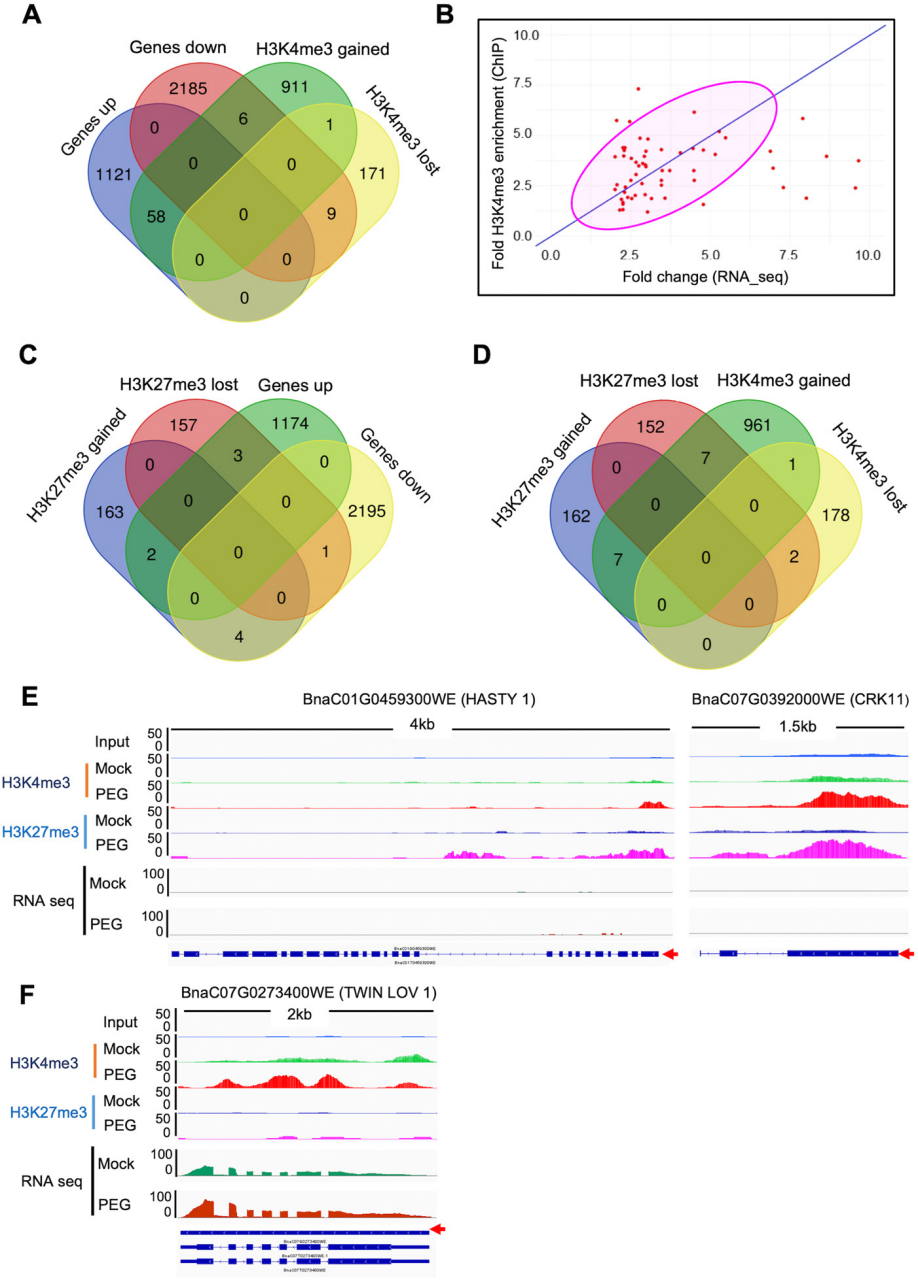
Integration of rapeseed ChIP-seq and RNA-seq data. (A) Venn diagram illustrating the overlap between H3K4me3 targets and DEGs in response to 24 h of PEG treatment. (B) Scatter plot visualization of the relationship between RNA-seq and ChIP-seq analysis for the 58 PEG-induced genes that gained H3K4me3 marks. (C, D) Venn diagrams showing the overlap between H3K27me3 targets and DEGs in response to 24 h of PEG treatment (C) and the intersection between genomic loci marked by H3K4me3 and H3K27me3. (E) ChIP-seq and RNA-seq tracks showing H3K4me3 and H3K27me3 chromatin marks at two representative genes: *HASTY 1* and *CRK11*. (F) ChIP-seq and RNA-seq tracks of the gene *TWIN LOV 1* gene showing differential enrichment of H3K4me3 chromatin marks upon PEG treatment.

To investigate whether gene expression patterns correlate with histone mark distributions, we conducted a comparative analysis of RNA-seq and ChIP-seq profiles. Among the 1179 genes up-regulated after 24 h of PEG treatment, 58 genes exhibited an increase in the active H3K4me3 marks (Fig. 6A; Supplementary Dataset S13). For example, *BnRAB18*, *BnPP2C*, and *BnH2A7* genes were up-regulated upon PEG treatment and had H3K4me3 enrichment (Supplementary Fig. S12). Scatter plot analysis for these 58 genes revealed a correlation of change in gene expression and increase in H3K4me3 binding, suggesting that augmented H3K4me3 binding contributes to increased gene expression (Fig. 6B). A heatmap illustrated expression patterns of these 58 genes with increased H3K4me3 marks (Supplementary Fig. S13). Among the 2200 genes down-regulated under the same conditions, six showed a gain and nine showed a loss of H3K4me3 marks (Fig. 6A).

Many PEG-induced genes with increased H3K4me3 marks are implicated in stress responses and include those encoding transcription factors (ABF3, ABI5-like, GBF3, and HSFA6A), proteins involved in signal transduction (ABI2/PP2C protein phosphatases and RLK-like kinase), heat shock proteins and chaperones (HSP40 and BCS1-like mitochondrial chaperone), E3 ubiquitin-protein ligase, several dehydrins, and LEA-type proteins (LTI65, RAB18, and GMPM1/LEA). Two PEG-induced *P5CSA* genes could be identified among those which had enhanced H3K4me3 marks (*BnP5CSA.3* and *BnP5CSA.5*), suggesting that the rate-limiting step in proline biosynthesis is under epigenetic regulation (Supplementary Fig. S13).

Two and three genes had increased and decreased H3K27me3 marks among the up-regulated genes, respectively. Among the 2200 PEG-repressed genes, four genes gained and one gene lost the H3K27me3 mark (Fig. 6C). Although H3K27me3 and H3K4me3 modifications are traditionally considered counterbalancing and mutually exclusive (Schwartz and Pirrotta, 2007; Bouyer *et al.*, 2011; Roudier *et al.*, 2011), our findings suggest that the levels of H3K27me3 and H3K4me3 can be independent of each other’s presence. While H3K4me3 levels showed some correlation with transcription of many stress-responsive genes, H3K27me3 levels remained constant regardless of their transcriptional status, indicating that these two histone marks do not counterbalance each other.

Differential enrichment of H3K4me3 and H3K27me3 marks is shown on two representative genes: *BnHASTY1-like* and *BnCRK11* (Fig. 6E). HASTY 1 is known for its role in miRNA biogenesis and for mediating the export of miRNAs from the nucleus to the cytoplasm. Cysteine-rich receptor-like kinases (CRKs), such as CRK11, control disease resistance and cell death in plants. Our study revealed that both H3K4me3 and H3K27me3 marks were present on these genes (Supplementary Dataset S14). There was no PEG-dependent change in their expression levels, therefore the function of these marks remains unclear. The genomic coding region of the PEG-induced *TWIN LOV 1* had both gain and loss of H3K4me3 marks in different regions (Fig. 6F).

We compared the transcriptomic and epigenetic profiles of *B. napus* with those of its diploid progenitors. A total of 100 194 transcripts were identified in *B. napus*, with 43 246 (43.2%) derived from the A-subgenome, 50 845 (50.7%) from the C-subgenome, and 6103 (6.1%) originating from scaffold sequences (Supplementary Fig. S14A). A total of 24 508, 27 969, and 2353 genes were actively expressed in the A-, C-, and scaffold subgenomes, respectively (Supplementary Fig. S14B). Our transcriptomic analysis identified 5473 and 3379 DEGs in *B. napus* under PEG stress at 6 h and 24 h, respectively (Fig. 3C, D). Transcripts from both the A- and C-subgenomes had a similar distribution of expression levels at both 6 h and 24 h of PEG treatment (Supplementary Fig. S14C, D). Regions derived from the C-subgenome showed a higher enrichment of H3K4me3 marks compared with those from the A-subgenome (Supplementary Fig. S14E), while a greater number of regions with H3K27me3 loss were detected in the C-subgenome (Supplementary Fig. S14F).

To obtain a broader perspective on the integration of RNA-seq data with H3K4me3 enrichment, we reduced the stringency of the RNA-seq analysis. Using a log_2_ FC cut-off of 1.5 and a *P*adj value of 0.05, we identified a total of 5202 genes that were differentially expressed in response to 24 h PEG treatment. Of these, 1872 genes were up-regulated, while 3330 genes were down-regulated (Supplementary Fig. S15A). Employing a log_2_ FC cut-off of 1.0 and a *P*adj value of 0.05 led to the identification of 8149 DEGs, with 3123 up- and 5026 down-regulated genes (Supplementary Fig. S15B), Replicates showing deviations were excluded to ensure the reliability of the analysis. The complete list of DEGs is provided in Supplementary Dataset S15.

To further explore the relationship between gene expression and chromatin modifications, we integrated the RNA-seq data with H3K4me3 enrichment profiles. This analysis revealed 88 genes that were up-regulated with H3K4me3 gain, 10 genes that were down-regulated with H3K4me3 loss, and nine genes that were down-regulated but exhibited H3K4me3 gain (Supplementary Fig. S15C). The complete list of these genes, along with their detailed descriptions, is available in Supplementary Dataset S16.

### Epigenetic regulation of rapeseed *P5CS* genes

**Fig. 7.**
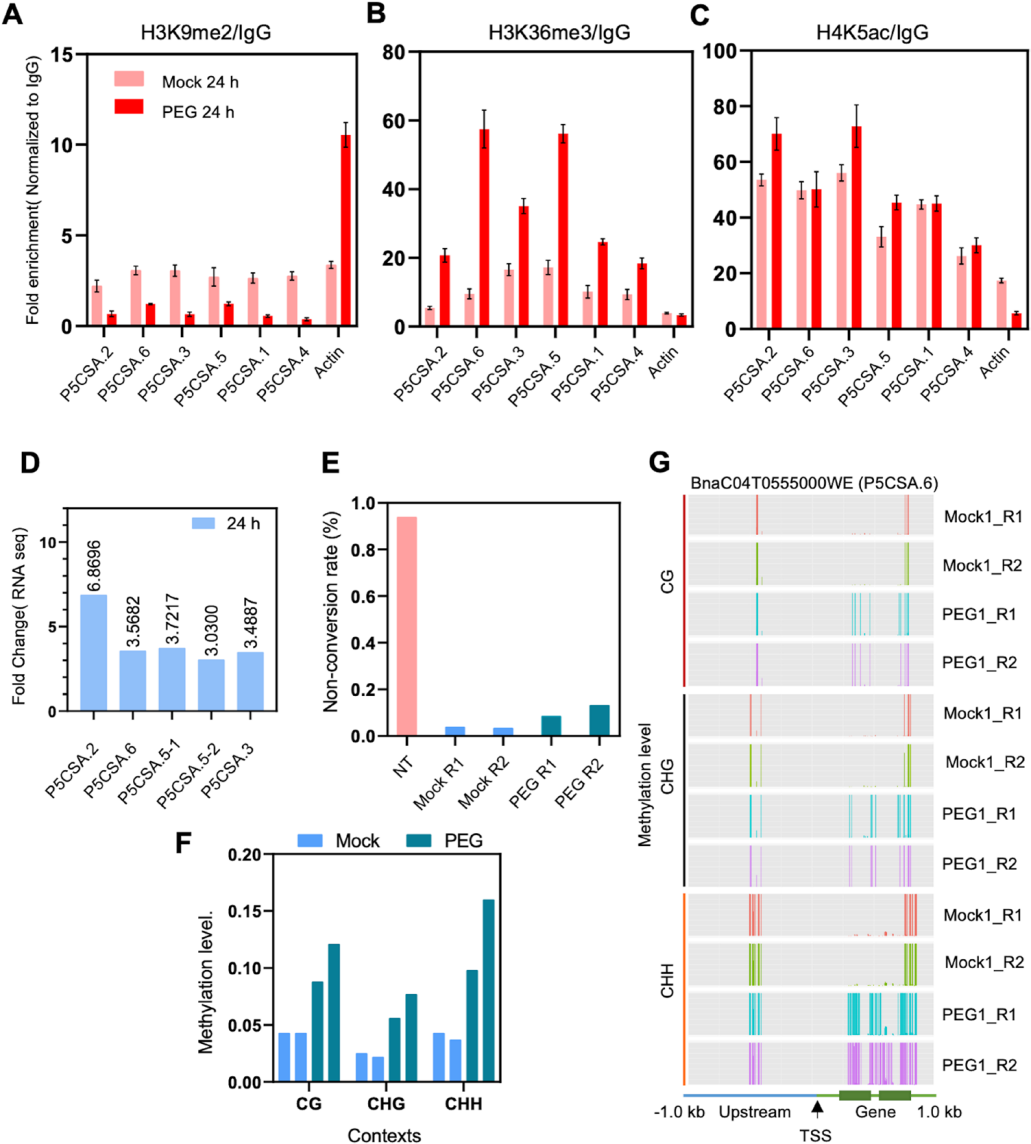
Epigenetic regulation of *P5CSA* in response to PEG treatment. (A–C) Differential enrichment patterns for H3K9me2 (A), H3K36me3 (B), and H4K5ac (C) at rapeseed *BnP5CSA* genes in response to 24 h of PEG treatment (ChIP-qPCR). (D) Enhancement of rapeseed *BnP5CSA* expression in response to 24 h of PEG treatment. (E) Non-conversion rate of rapeseed DNA isolated from Mock and PEG-treated samples. (F) Average DNA methylation levels at *BnP5CSA* loci in different cytosine contexts (CG, CHG, and CHH) in response to PEG treatment. (G) DNA methylation profile of *P5CSA.6* obtained from targeted bisulfite sequencing; 1 kb upstream and downstream regions from the transcription start site (TSS) are shown.

The rapeseed genome is more complex than that of Arabidopsis, which is reflected by the higher number of genes which encode key enzymes in proline metabolism (Supplementary Figs S2, S16; Supplementary Table S6). RNA-seq analysis indicates that most *BnP5CSA* genes and two out of the four *BnP5CSB* genes are induced by PEG treatment (Fig. 7D; Supplementary Fig. S16; Supplementary Table S6). Transcriptional activation correlated with the enrichment of the H3K4me3 activation mark in *BnP5CSA.3*, *BnP4CSA.5*, and *BnP5CSB.4* genes (Supplementary Fig. S16; Supplementry Table S6). Three out of the 14 identified *P5CR* genes had detectable transcripts, but were not significantly influenced by PEG. Fast activation of *BnPDH1.1* and *BnPDH1.4* was found after 6 h of PEG, while expression of *BnPDH2.1* was enhanced only after 24 h. Two out of the five *BnP5CDH* genes were expressed and could be slightly induced by PEG (*BnP5CDH.2* and *BnP5CDH.5*). None of these genes had differences in H3K4me3 or H3K27me3 marks (Supplementary Table S6).

To extend our studies to other histone modifications on *BnP5CS* genes, enrichments of H3K9me2, H3K36me3, and H4K5Ac marks were tested. ChIP-qPCR was performed using anti-H3K9me2, anti-H3K36me3, and anti-H4K5Ac antibodies targeting the TSS regions (Supplementary Table S2). Reduction in H3K9me2 was found across all *BnP5CSA* genes upon PEG treatment compared with the control, while the H3K36me3 activation mark was significantly enhanced in the PEG-treated samples (Fig. 7A, B). *BnP5CSA.1*, *BnP5CSA.3*, and *BnP5CSA.5* also showed enrichment of the H4K5Ac mark upon stress (Fig. 7C). Such trends were not observed in the actin gene.

### PEG-induced hypermethylation in a rapeseed *P5CSA* gene

DNA methylation plays a pivotal role in regulating numerous genes involved in stress responses. We were particularly interested in DNA methylation of stress-induced *BnP5CS* genes and selected the stress-induced *BnP5CSA.6* for further analysis (Fig. 7D; Supplementary Table S6). Targeted bisulfite sequencing covered 1 kb upstream and 1 kb downstream of the TSS and was performed on PEG-treated and control samples (Supplementary Table S3). PEG-treated samples showed a slightly higher non-conversion rate compared with control samples (Fig. 7E). Methylation analysis revealed a PEG-dependent increase in methylation levels across all sequence contexts, including CG, CHG, and CHH, in *BnP5CSA* genes (Fig. 7F). PEG-dependent hypermethylation was predominantly observed within the gene body of *BnP5CSA.6*, with significant enrichment at the TSS, exon 1, exon 2, and the intronic region between exon 1 and exon 2. Although some methylation was detected at –0.5 kb in the upstream region, a PEG-dependent change was not observed in the promoter of this gene (Fig. 7G). These data suggest that enhanced DNA methylation in the transcribed region of the *BnP5CSA.6* gene might have contributed to the increased expression.

## Discussion

### Physiological and molecular consequences of PEG treatment in rapeseed

Our study provides important insights into the complex responses of rapeseed to drought stress, revealing significant changes in gene expression and epigenetic regulation on a genomic scale. The dynamic nature of these adjustments is essential for plant survival in water-restricted conditions. Previous studies, predominantly performed on model plants, have emphasized the role of epigenetic mechanisms, such as DNA methylation and histone modifications, in plant responses to abiotic stresses (Nunez-Vazquez *et al.*, 2022; Sun *et al.*, 2022). However, information on the specific roles of chromatin modifications in non-model plants in water-restricted conditions is scarce (Thiebaut *et al.*, 2019). Transcriptional responses of salt- and drought-treated rapeseed have been investigated by Wang *et al.* (2019), although the conditions were quite different. Through the integration of ChIP-seq and RNA-seq, we have identified gene sets in rapeseed that respond to osmotic stress, highlighting the epigenetic regulation involved.

PEG-induced osmotic stress can mimic a water-restricted environment which generates physiological and developmental responses similar to water shortages during drought (Verslues *et al.*, 2006). In rapeseed, we observed substantial morphological, physiological, and biochemical changes in response to PEG. One response was proline accumulation, a common physiological response to osmotic and salinity stress, which is a sensitive indicator of stress conditions (Fig. 1; Supplementary Fig. S2). Proline plays a role in osmoregulation, maintenance of cellular homeostasis, and redox balance (Szabados and Savouré 2010; Patriarca *et al.*, 2021; Alvarez *et al.*, 2022). PEG enhanced proline levels in rapeseed leaves after 24 h, followed by accumulation up to 7 d of stress (Fig. 1). In a transcriptomic study, Wang *et al.* (2019) reported an ~2-fold proline accumulation in rapeseed seedings after 3 h of PEG treatment. Our results somehow contradict that report, as a difference in proline content could not be observed in rapeseed after 6 h of PEG treatment (Fig. 1).

Altered physiological parameters such as ROS accumulation, enhanced lipid peroxidation, and increased leaf temperature indicated the damaging effect of PEG treatment which reduced photosynthetic activity (Fig. 1) and probably affected carbon assimilation (Buchert *et al.*, 2021; Hikosaka and Tsujimoto, 2021). Activation of rapeseed homologues of Arabidopsis stress- and ABA-induced genes (Fig. 2) indicated that PEG treatment triggered stress regulatory mechanisms similar to the well-characterized Arabidopsis signalling pathways (Strizhov *et al.*, 1997; Sakuma *et al.*, 2006; Matsui *et al.*, 2008; Takahashi *et al.*, 2018). At*NCED3* controls the rate-limiting step of ABA synthesis in Arabidopsis (Iuchi *et al.*, 2001; Finkelstein, 2013). Up-regulation of *BnNCED3* in PEG-treated rapeseed indicated enhanced ABA biosynthesis, which could be responsible for the enhanced transcript levels of the ABA-responsive genes *BnP5CSA*, *BnRAB18*, *BnRD29A*, and *BnRD29B* (Fig. 2).

### RNA-seq analysis reveals large-scale changes in transcript profiles of PEG-treated rapeseed

In order to identify gene sets that respond to osmotic stress in rapeseed, transcript profiling was carried out after 6 h and 24 h of PEG treatment. Initial PCA results pointed to outliers in 24 h RNA-seq samples (Supplementary Fig. S3). Eliminating outlying replicates led to more consistent datasets confirmed by better PCA (Fig. 3A). The numbers of identified DEGs (Fig. 3; Supplementary Fig. S3) were comparable with earlier studies, performed on drought- or salt-stressed rapeseed plants (Yong *et al.*, 2014; Liu *et al.*, 2015; Wang *et al.*, 2017). A lower number of DEGs was identified by Wang *et al.* (2017), who defined 169 genes to be crucial in response to drought. In contrast, we found found 5473 DEGs after 6 h and 3379 DEGs after 24 h of PEG treatment (Fig. 3). The number of DEGs was lower after 24 h of PEG treatment, suggesting that fast signal transduction and an early transcriptional response to osmotic stress are critical in rapeseed. A total of 913 DEGs were identified in rapeseed after 3 h of PEG treatment, which was, however, insufficient to enhance ROS levels and oxidative damage (Wang *et al.*, 2019). Significantly altered physiological parameters and the high number of DEGs in transcript profiling indicated a more robust osmotic stress in our experiments.

Genes implicated in stress responses, defence, and various metabolic processes were up-regulated, while genes involved in photosynthesis, protein biosynthesis, and membrane transport processes were down-regulated under osmotic stress. Some differences in fast and long-term responses could be observed in the DEG profiles (Supplementary Figs S4, S5). Genes differentially regulated at 6 h and 24 h of PEG treatment (Fig. 3) may correlate with the alterations in GO term profiles at these time points. In plants, various classes of transcription factors are activated in response to drought, orchestrating complex gene expression changes. We observed activation of various transcription factor families, including DREB, ABI/ABF, bZIP, HD-ZIP, MYB, NAC, and MYC-type transcription factors (Supplementary Fig. S6), probable members of gene regulatory networks which control stress responses.

The activites of genes encoding chromatin regulators were also induced by PEG, including *BnDRM2*, *BnSUVH6*, *BnHDAC*, *BnJMJ*, *BnRDR5*, *BnATX1*, and *BnSUVH1*, while the transcript abundance of *BnMET1*, *BnCLASSY*, *BnHAC1*, *BnROS1*, and *BnDME* was reduced (Supplementary Fig. S7). Altered abundance of such chromatin regulators could modify the balance between methylated and unmethylated DNA, and acetylated or methylated histones (Penterman *et al.*, 2007; Torres and Fujimori, 2015). Altered chromatin structure influences DNA accessibility to transcription factors and RNA polymerases, and modulates gene expression in stress conditions (Luo *et al.*, 2012; Bhadouriya *et al.*, 2021).

### Osmotic stress generates large-scale modifications of epigenetic marks in rapeseed

Extensive DNA and histone modifications modulate gene expression in plants under stress conditions and can be reset to the basal level after recovery or sustained to generate stress memory to be transmitted to progenies (Chinnusamy and Zhu, 2009; Lämke and Bäurle, 2017). The role of histone methylation in regulating responses to environmental changes in plants has received attention in recent years (Abdulraheem *et al.*, 2024; Shi *et al.*, 2024), which is however poorly understood in rapeseed. We used a quantitative, single-base resolution technique (ChIP-seq) to profile genome-wide patterns of H3K4me3 and H3K27me3, two key histone modifications involved in stress-induced gene activation and repression, respectively (Kim *et al.*, 2015; Shi *et al.*, 2024). H3K4me3 was mainly located within genic regions, whereas H3K27me3 was significantly enriched within intergenic regions including TEs (Fig. 4). In PEG-treated rapeseed, nearly six times more H3K4me3 marks could be identified when compared with H3K27me3 marks (976 versus 169), while the numbers of lost H3K4me3 and H3K27me3 marks were similar in affected genes (181 versus 161) (Fig. 5). Such a difference suggests that the influence of H3K4me3 on gene activation is dominant over that of H3K27me3 in osmotically stressed rapeseed. A similar trend was reported in Arabidopsis, where H3K4me3 correlated with drought-dependent activation of stress-induced genes (Kim *et al.*, 2008). H3K4me3 and H3K27me3 often act antagonistically in plants exposed to environmental stress (Kim *et al.*, 2015). Differential histone methylation occured on distinct gene sets in cold-stressed Arabidopsis as H3K4me3 marks predominantly targeted stress-responsive genes, while H3K27me3 was primarily associated with developmental genes (Zeng *et al.*, 2019). In Arabidopsis, H3K4me3 and H3K27me3 marks can function independently and are not mutually exclusive at the dehydration stress-responsive memory genes (N. Liu *et al.*, 2014).

PEG-dependent accumulation of the H3K27me3 mark was observed in a small number of rapeseed genes, and included TRANSPARENT TESTA-12 like, translation initiation factor IF-3-like, AP2-like ethylene-responsive transcription factor, autophagy-related protein, MADS-box protein CMB1-like, and cytosolic sulfotransferase 16-like genes. Genes encoding disease resistance protein TAO1-like, ethylene-responsive transcription factor 1A-like, and PIF3 lost the H3K27me3 marks upon PEG treatment (Supplementary Dataset S12). ChIP-seq analysis revealed that the H3K27me3 mark is mostly distributed in the TE regions, encoding for LTR/Copia, LTR/Gypsy, DNA/CMC-EnSpm, and LINE/L1 transposons (Supplementary Figs S10, S11). Further analysis of such TE transcripts is needed to understand the influence of PEG stress on the regulation of TEs.

Despite generally distinct genomic locations and functions of the two histone modifications, we identified seven regions with H3K4me3 and H3K27me3 marks, including *BnHASTY1*, *BnWRKY19*, *BnCRK11*, and *BnDEGP14/PARK13*, which had lower transcript abundance after PEG treatment. The *BnTWIN LOV 1* gene (encoding a blue light photoreceptor protein) had increased H3K4me3 abundance within the gene body after stress (Fig. 6).

While thousands of DEGs were identified in PEG-treated samples and more than a thousand regions were found with stress-dependent enrichment differences of H3K4me3 or H3K27me3 marks, overlap between the two gene sets was moderate. We identified 58 PEG-induced genes with active H3K4me3 marks (Fig. 6; Supplementary Fig. S13). Many stress-related genes (*BnP5CS*, *BnRAB18*, *BnABI5*, Dehydrins, *BnRD23D*, Histone H2A7, RMA3-like, protein phosphatase 2C, and *BnHSF6*) had higher expression and were enriched with H3K4me3. Such enriched H3K4me3 marks could contribute to enhanced expression of the stress-responsive genes and facilitate the adaptation to drought. Overlap between DEGs and H3K27me3 targets was minimal, suggesting that this histone modification has no importance in PEG-dependent gene regulation (Fig. 6). The fact that only a small number of stress-induced genes were associated with H3K4me3 or H3K27me3 changes can be explained by the high stringency used in our study (at least 2-fold changes). With lower stringency (FC 1.5 and *P*adj 0.05), slightly more PEG-induced genes (88) could be identified with gained H3K4me3 marks (Supplementary Fig. S15). A recent report suggests that fast changes in gene activation/repression might not be associated with altered histone marks, which can further explain our observations (Holger and Deal, 2025, Preprint).

The allotetraploid *B. napus* (AACC) originated from the hybridization of two diploid species, *B. rapa* (2*n*=20, AA) and *B. oleracea* (2*n*=18, CC). Previous studies pointed to asymmetric genomic expression in such polyploids (S. Liu *et al.*, 2014). As *B. napus* is allopolyploid, the two subgenomes have undergone biased segregation following polyploidization and asymmetric genome evolution, including the asymmetric loss of subgenomes, differential TE amplification, and variations in DNA sequence and expression (S. Liu *et al.*, 2014; Bottani *et al.*, 2018). Polyploidization, biased segregation, and varying degrees of gene loss altered the composition of gene families, and contributed to the morphological plasticity in *Brassica* species (Wang *et al.*, 2011; Wendel *et al.*, 2018). We observed no significant differences in the distribution of PEG-dependent DEGs in the A- and C-subgenomes. However, more H3K4me3 and H3K27me3 marks could be found in the C-subgenome that were altered under stress conditions. These findings suggest that in the Westar variety of *B. napus* the C-subgenome may experience a higher frequency of epigenetic modifications and maintain greater nucleotide diversity compared with the A-subgenome (Supplementary Fig. S14).

### Epigenetic control of *P5CS* genes in rapeseed during osmotic stress

P5CS is a rate-limiting enzyme for proline synthesis, and is encoded by two genes in Arabidopsis, the stress-induced *P5CS1* and the housekeeping *P5CS2* (Yoshiba *et al.*, 1995; Savouré *et al.*, 1997; Strizhov *et al.*, 1997; Székely *et al.*, 2008). We have identified 10 *P5CS* genes in rapeseed, grouped into two subfamilies, *BnP5CSA* and *BnP5CSB* (Supplementary Fig. S16; Supplementary Table S6), and showed that these genes were up-regulated by PEG-generated osmotic stress (Fig. 2). RNA-seq analysis revealed that four *BnP5CSA* genes and two *BnP5CSB* genes were induced by 6 h and 24 h PEG treatments, while the others were not activated (Supplementary Fig. S16; Supplementary Table S6). ChIP-seq analysis revealed that regions encoding *BnP5CSA.3*, *BnP5CSA.5*, and *BnP5CSA.4* genes had enriched H3K4me3 marks upon PEG treatment (Supplementary Table S6). H3K9me2, H3K36me3, and H4K5Ac marks can also influence the activity of *BnP5CSA* genes (Fig. 7). H3K9me2 is associated with transcriptional repression in animal cells (Padeken *et al.*, 2022), which was reduced in *BnP5CSA* genes under stress. In contrast, H3K36me3 levels were enriched at the TSSs, while H4K5Ac was altered on three *BnP5CSA* genes. Our transcriptome analysis identified differential expression of various chromatin regulators under stress conditions (Supplementary Fig. S7), which can modulate histone acetylation and methylation of various *BnP5CS* genes. *BnP5CR*, *BnPDH1*, *BnPDH2*, and *BnP5CDH* genes had detectable transcript levels, some of them with PEG-dependent activation. None of these genes, however, had altered histone modifications (Supplementary Table S6), suggesting that chromatin methylation has no influence on their expression.

Transcript mapping of *BnP5CSA.5* in the RNA-seq experiment could identify three transcripts of this gene (Supplementary Fig. S16; Supplementary Table S6), suggesting that alternative splicing might regulate at least one *BnP5CS* gene in rapeseed. Intron-mediated alternative splicing was shown to regulate *P5CS1* activity in Arabidopsis as well (Kesari *et al.*, 2012). Alternative splicing can therefore control the activity of some of the *P5CS* genes in *Brassicaceae* species.

DNA methylation is a hallmark epigenetic modification that plays a crucial role in regulating genome stability, chromatin structure, gene expression, and silencing of TEs in extreme environments. In plant genes, the effect of DNA methylation depends on the position: in promoters, it usually inhibits gene expression, while in exons CG methylation may enhance transcription (Lei *et al.*, 2015; Thiebaut *et al.*, 2019; Sun *et al.*, 2022). In Arabidopis, gene body methylation is typically restricted to the CG context. However, in other plants species, CHH and CHG methylation may also occur in specific key genes (Fan *et al.*, 2020; Miryeganeh, 2021; Y. Wang et al., 2021; Sun *et al.*, 2022), presenting an interesting avenue for further study. Our analysis focused on the transcribed region of the PEG-induced *BnP5CSA.6* gene, which had enrichment in H3K36me3 marks upon osmotic stress (Fig. 7; Supplementary Table S6). We found hypermethylation across all contexts (CG, CHG, and CHH) within exon 1 and exon 2 in PEG-treated samples, indicating that DNA methylation might influence expression of this gene. Recent reports suggest that gene body methylation can be associated with elevated gene expression and plasticity of gene activation (Muyle *et al.*, 2022; Williams *et al.*, 2023). DNA hypermethylation has been shown to influence gene expression under various stress conditions, including up-regulation and stabilization of transcription (Chinnusamy and Zhu, 2009; Muyle *et al.*, 2022; Sun *et al.*, 2022). Dynamic DNA methylation of *BnP5CSA.6* may therefore contribute to its activation in PEG-treated rapeseed plants. The down-regulation of *BnDME* and *BnROS1* DNA demethylases observed in the transcriptome data (Supplementary Fig. S7) might contribute to *BnP5CSA.6* hypermethylation under PEG-induced stress (Fig. 7). In Arabidopsis, increased H3K4me3 levels of the stress-induced *P5CS1* gene were maintained after stress recovery and contribute to transcriptional memory (Feng *et al.*, 2016). In drought-stressed tomato roots, decreased CHH methylation was found in regulatory regions, but increased CHG and CHH methylation was observed in coding regions of the ABSCISIC ACID STRESS RIPENING 2 (*SlAsr2*) gene (González *et al.*, 2013). DNA hypermethylation in salt-tolerant genotypes of rice was also observed, while sensitive genotypes exhibited demethylation (Feng *et al.*, 2012). Such findings underscore the potential regulatory roles of DNA methylation of transcribed regions (Muyle *et al.*, 2022; Sun *et al.*, 2022).

In conclusion, our study reveals that large-scale transcriptomic changes and epigenetic modifications can take place in drought-stressed rapeseed plants. The generated expression and epigenetic profiles of rapeseed can facilitate the understanding of gene regulatory mechanisms on a genomic scale in such allotetraploid crops with a complex genome. Characterized epigenetic marks can be targets of genome editing with DNA modifiers or chromatin remodellers to edit epigenetic marks in crops such as rapeseed and to engineer gene expression profiles to improve drought tolerance (Selma and Orzáez 2021; Seem *et al.*, 2024).

## Supplementary data

The following supplementary data are available at *JXB* online.

Fig. S1. Response of rapeseed to osmotic stress.

Fig. S2. Schematic model of proline metabolism in plants.

Fig. S3. Analysis of RNA-seq data with two or three replicates.

Fig. S4. GO analysis of differentially regulated genes after 6 h of PEG treatment.

Fig. S5. GO analysis of differentially regulated genes after 24 h of PEG treatment.

Fig. S6. Heatmap of differentially expressed transcription factors.

Fig. S7. Heatmap of differentially expressed chromatin remodellers.

Fig. S8. Hierarchical clustered correlation matrix for ChIP-seq profiling data.

Fig. S9. ChIP-seq analysis of rapeseed in response to PEG treatment.

Fig. S10. Transposons showing differential enrichment of H3K4me3 marks.

Fig. S11. Transposons showing differential enrichment of H3K27me3 marks.

Fig. S12. ChIP-seq and RNA-seq tracks in three selected stress genes.

Fig. S13. Heatmap of the RNA-seq and ChIP-seq data of 58 selected genes.

Fig. S14. Asymmetrical distribution of epigenomic marks in subgenomes of *B. napus*.

Fig. S15. Integration of rapeseed ChIP-seq and RNA-seq data with reduced stringency.

Fig. S16. H3K4me3 and H3K27me3 enrichments of *BnP5CSA* and *BnP5CSB* genes.

Table S1. Primers for RT–qPCR analysis of stress-responsive genes in *Brassica napus*.

Table S2. Primers for ChIP-RT–qPCR analysis of *BnP5CSA* genes.

Table S3. Primers for targeted bisulfite sequencing analysis of *BnP5CSA* genes.

Table S4. RNA-seq quality metrics.

Table S5. ChIP-seq quality metrics.

Table S6. Transcript and histone modification data of proline metabolic genes.

Dataset S1. DEGs at 24 h, including outliers (FC 2.0, *P*adj 0.05).

Dataset S2. Common and unique RNA-seq DEGs at 24 h with two or three replicates.

Dataset S3. DEGs at 6 h (FC 2.0, *P*adj 0.05).

Dataset S4. DEGs at 24 h (FC 2.0, *P*adj 0.05).

Dataset S5. All RNA transcript counts at 6 h and 24 h.

Dataset S6. PEG-induced DEGs at 24 h versus 6 h (FC 2.0, *P*adj 0.05).

Dataset S7. DEGs up-regulated at 6 h and 24 h (FC 2.0, *P*adj 0.05).

Dataset S8. DEGs down-regulated at 6 h and 24 h (FC 2.0, *P*adj 0.05).

Dataset S9. DEGs induced at 6 h, down-regulated at 24 h (FC 2.0, *P*adj 0.05).

Dataset S10. DEGs down-regulated at 6 h, induced at 24 h (FC 2.0, *P*adj 0.05).

Dataset S11. H3K4me3-enriched regions.

Dataset S12. H3K27me3-enriched regions.

Dataset S13. H3K4me3 enriched, 58 induced genes at 24 h (FC 2.0, *P*adj 0.05).

Dataset S14. Simultaneous H3K4me3- and H3K27me3-enriched regions.

Dataset S15. DEGs at 24 h excluding outliers (FC 2.0, *P*adj 0.05).

Dataset S16. H3K4me3-enriched, DEGs at 24 h (FC 2.0, *P*adj 0.05).

eraf123_suppl_Supplementary_Figures_S1-S16

eraf123_suppl_Supplementary_Tables_S1-S6

eraf123_suppl_Supplementary_Datasets_S1-S16

## Data Availability

The data supporting the findings of this study are available upon request from the corresponding author, LS (szabados.laszlo@brc.hu). The raw sequencing data have been deposited in the NCBI and can be accessed at: RNA-seq, https://dataview.ncbi.nlm.nih.gov/object/PRJNA1144338?reviewer=60o8cel20hm9nhfovce9oitou5; ChIP-seq, https://dataview.ncbi.nlm.nih.gov/object/PRJNA1144218?reviewer=qnlmhdp73uksjnvl3ju8jgv18e; and targeted bisulfite sequencing, https://dataview.ncbi.nlm.nih.gov/object/PRJNA1144360?reviewer=t3v21vbt3fpdem9f12100qgsu.
