# Supplementary material for: Transcriptional and epigenomic changes in response to polyethylene glycol-triggered osmotic stress in Brassica napus L
Source: J Exp Bot. 2025 Apr 3;76(9):2535–56. doi: 10.1093/jxb/eraf123 (PMC12192427; doi:10.1093/jxb/eraf123)
Supplement: eraf123_suppl_Supplementary_Figures_S1-S16 [file eraf123_suppl_supplementary_figures_s1-s16.pdf]

Supplementary figures

Transcriptional and epigenetic response of rapeseed (*Brassica napus* L.) to PEG-triggered osmotic stress.

Melvin Prasad*[1], Prateek Shetty[1], Avik Kumar Pal[2], Gábor Rigó[1], Kamal Kant[1],Laura Zsigmond[1], István Nagy[3], P. V. Shivaprasad[2] and László Szabados*[1]

[1]*Institute of Plant biology, HUN-REN BRC, Szeged, Hungary*

[2]*National Centre for Biological Sciences (NCBS)- Tata Institute of Fundamental Research (TIFR), Bangalore, India*

[3]*Seqomics Kft, Mórahalom, Hungary*

- *Correspondence: szabados.laszlo@brc.hu, melvinp@brc.hu and melvinprasad@gmail.com*

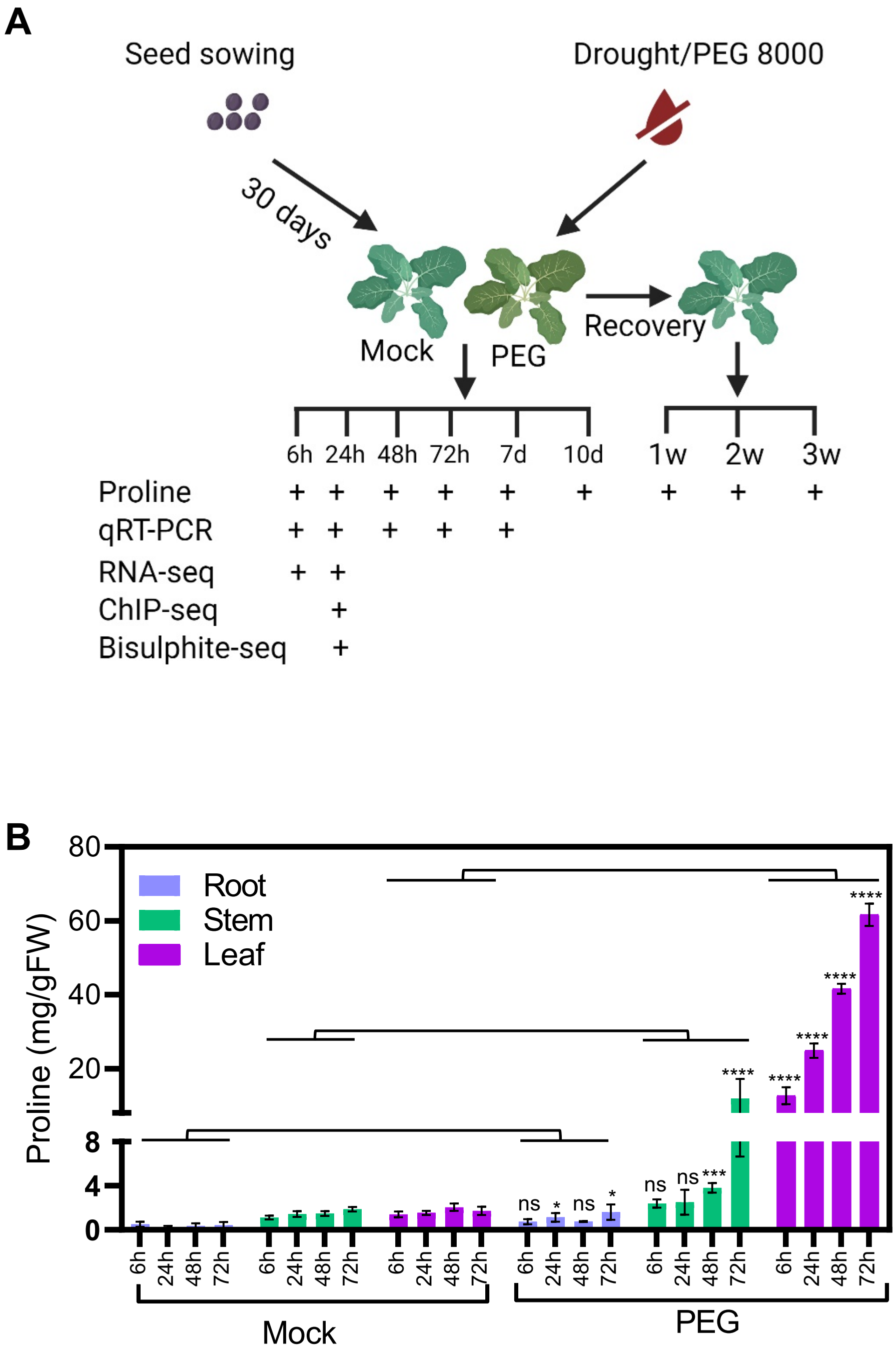

Figure S1: Response of rapeseed to osmotic stress. **A)** Overview of Experimental Design: Schematic representation illustrating the experimental workflow. **B)** Proline accumulation in different rapeseed organs (roots, stems, and leaves) exposed to 20 % PEG for 24, 48, and 72 h. Bars represent the mean ± SE of three independent experiments. Statistical analysis was performed using a two-way ANOVA followed by Tukey's multiple comparison test. Columns labeled with "ns" indicate no significant difference, while asterisks (*) denotes $p < 0.05$, (**) $p < 0.01$, (***) $p < 0.001$, and (****) $p < 0.0001$ indicate statistically significant differences among treatments.

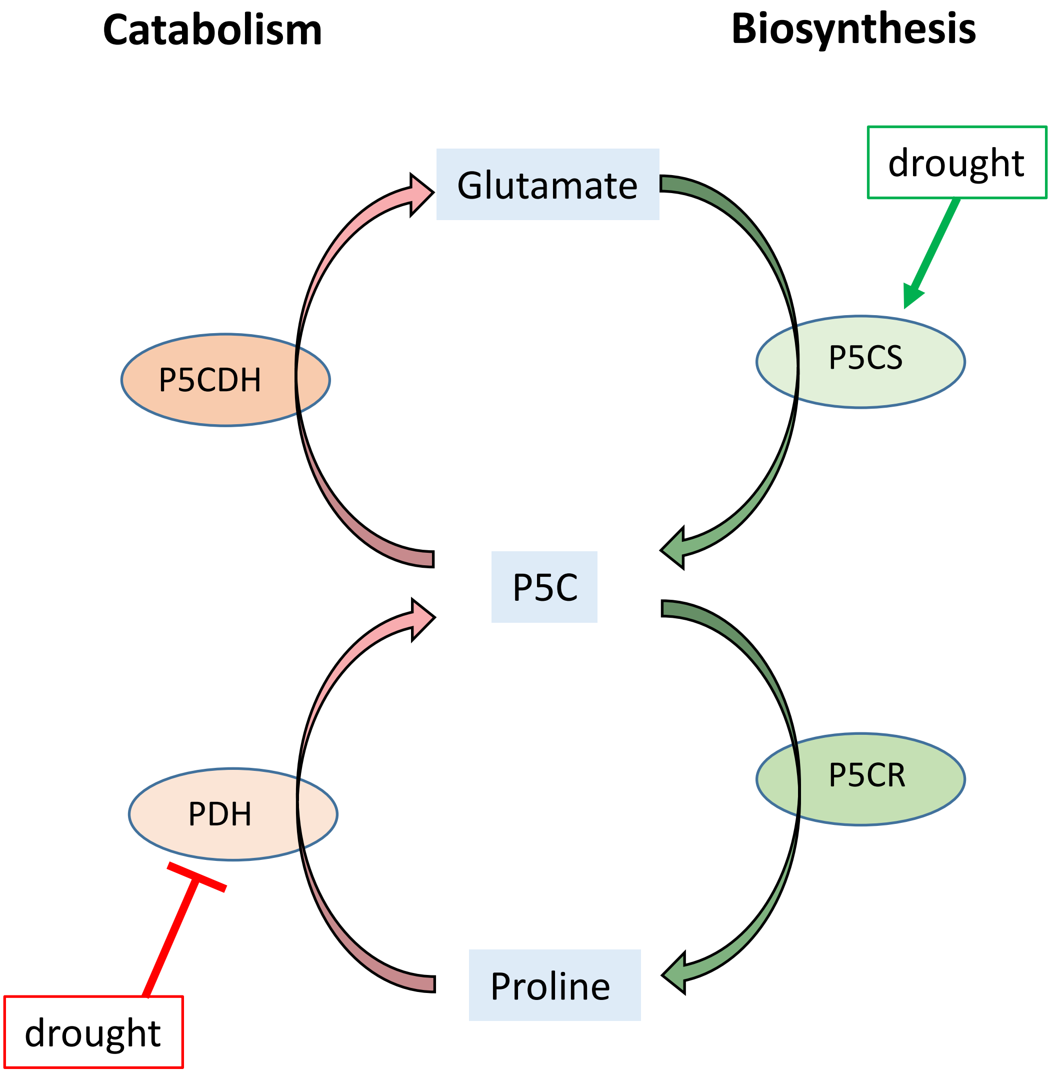

Abbreviations:
P5CS: Δ[illegible]-pyrroline-5-carboxylate synthase
P5CR: Δ1-pyrroline-5-carboxylate reductase
PDH: Proline dehydrogenase
P5CDH: Δ[illegible]-pyrroline-5-carboxylate dehydrogenase
P5C: pyrroline-5-carboxylate

Figure S2: Schematic model of proline metabolism in plants. Proline is synthetized mainly from glutamate in two steps, catalized by the P5CS and P5CR enzymes. Proline is degraded in the mitochndria, by stevise activities of PDH and P5CDH enzymes (Szabados and Savoré, 2010, Alvarez et al., 2022).

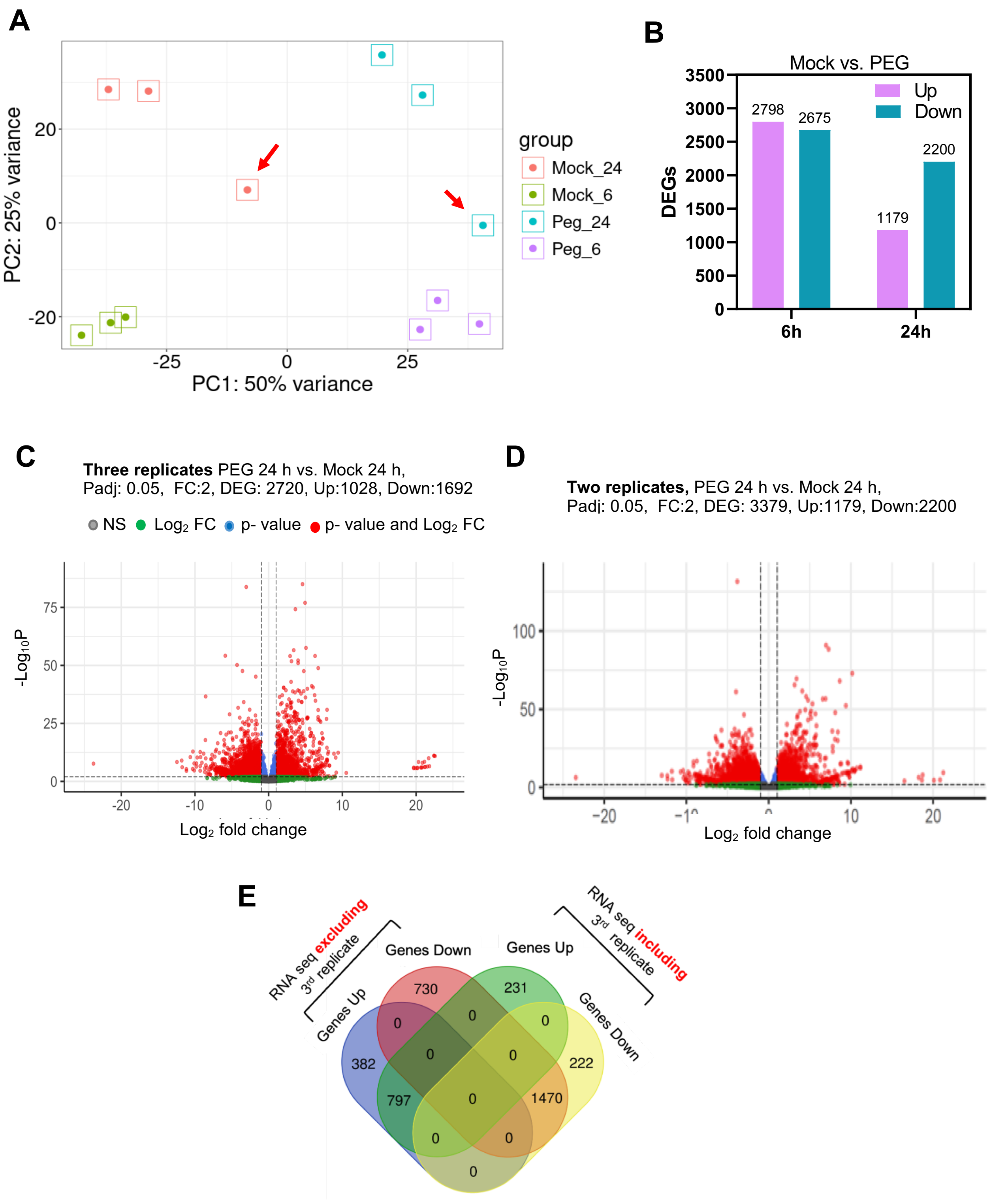

Figure S3: Analysis of RNAseq data with two or three replicates. A) Principal Component Analysis (PCA) of transcriptomic datasets from Mock and PEG-treated plants (3 biological replicates each). Arrows indicate replicates that deviate from their respective groups. **B**) Representation of the number of up and downregulated rapeseed genes after 6 h or 24 h of PEG treatment. **C,D**) Volcano plot Analysis of differentially expressed genes (DEGs) in Mock and PEG-treated samples after 24 h of PEG treatment, using three replicates **(C)** or two replicates excluding the outlier groups (D). **E)** Venn diagram depicting the overlap between differentially expressed genes (DEGs) identified with and without the inclusion of the third replicate in response to 24-hour PEG treatment (Fold Change: 2.0, Padj: 0.05)

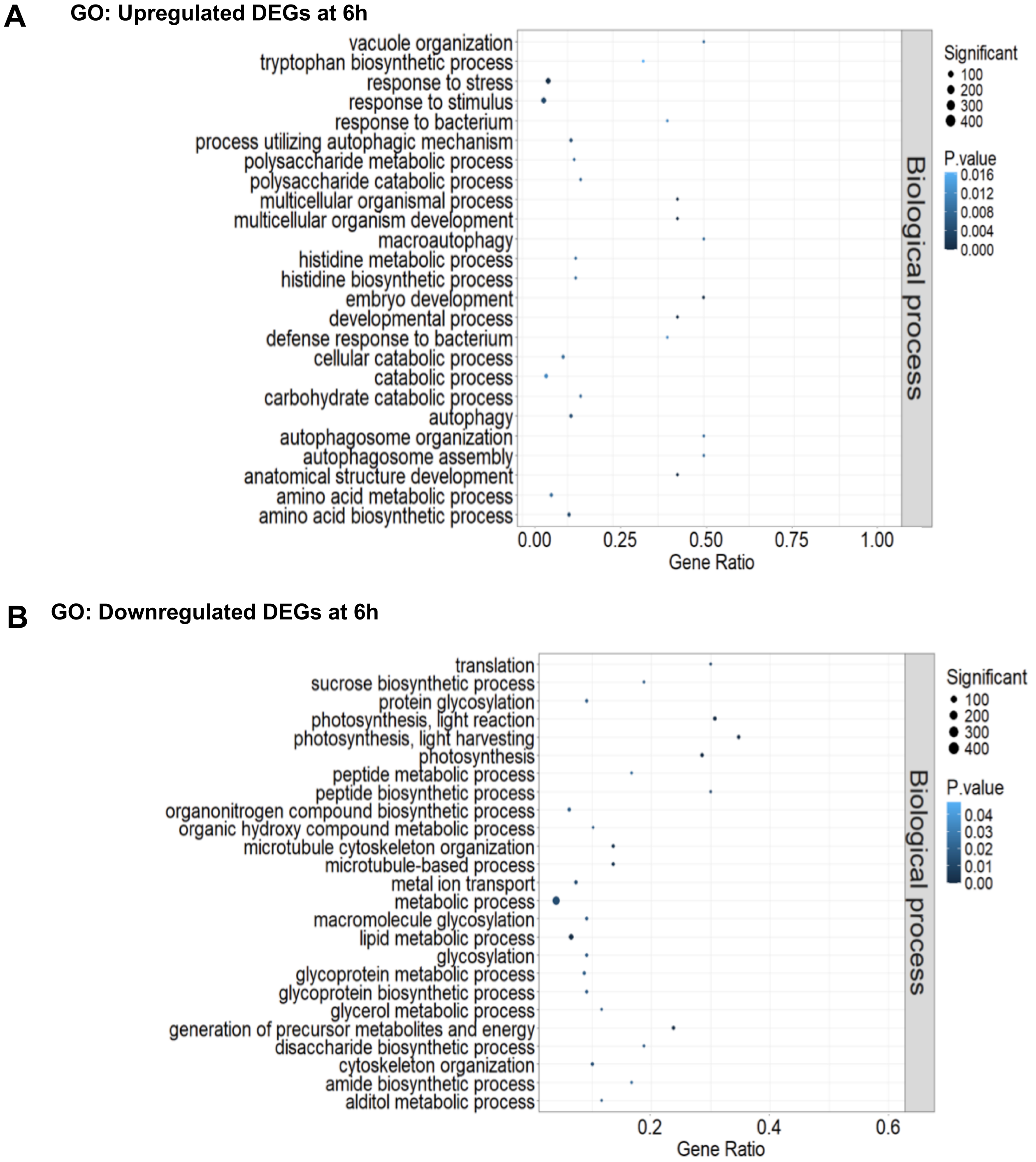

Figure S4. Gene Ontology analysis of differentially regulated genes after 6h of PEG treatment. Functional categorization of DEGs with a False Discovery Rate (FDR) q-value less than 0.05. GO terms of biological processes are displayed. **A)** Upregulated genes. **B)** Downregulated genes.

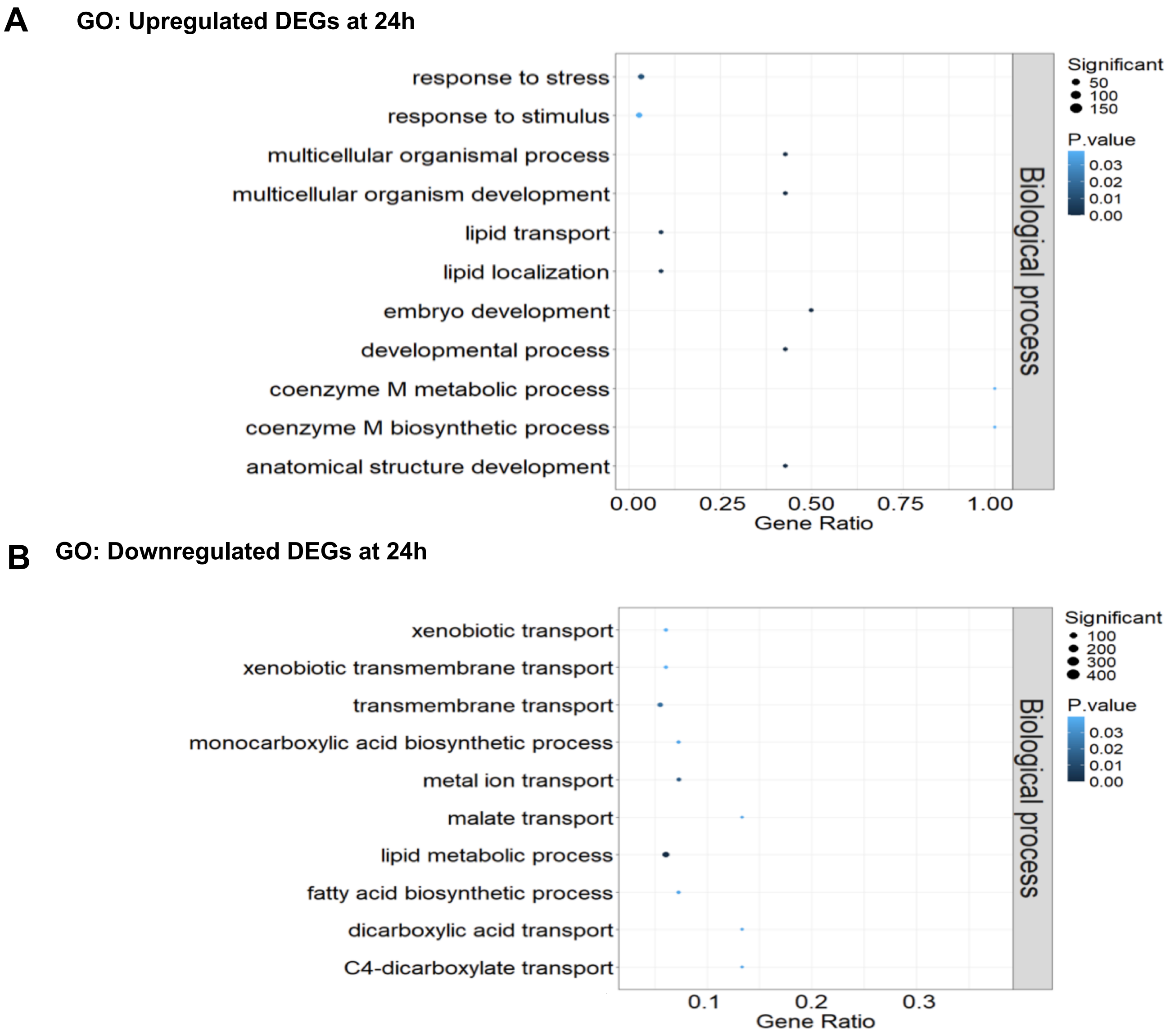

Figure S5. Gene Ontology analysis of differentially regulated genes after 24 h of PEG treatment. Functional categorization of DEGs with a False Discovery Rate (FDR) q-value less than 0.05. GO terms of biological processes are displayed. **A)** Upregulated genes. **B)** Downregulated genes.

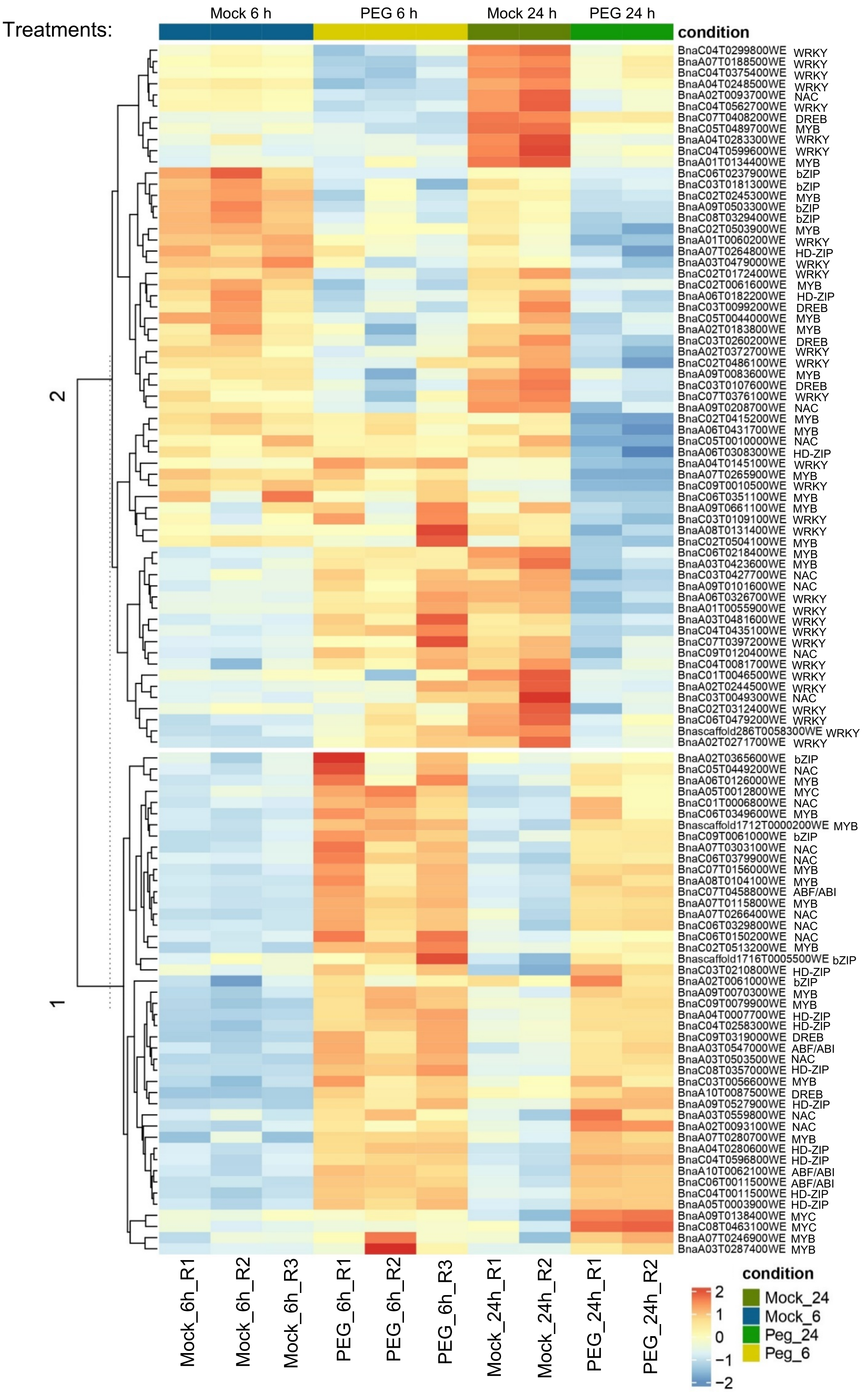

Figure S6: Heatmap of differentially expressed transcription factors after 6 h or 24 h of PEG treatment.

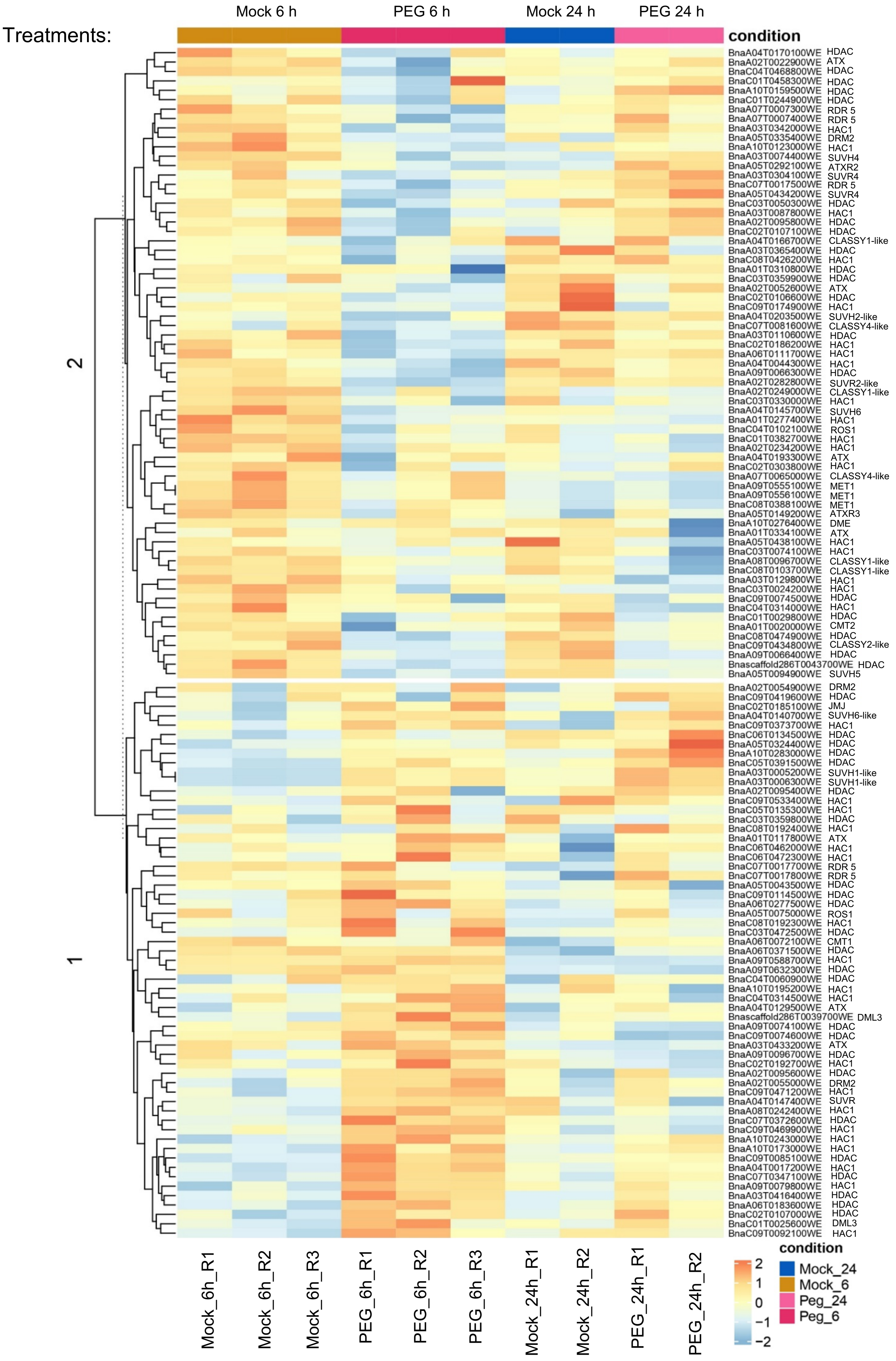

Figure S7: Heatmap of differentially expressed chromatin regulating genes after 6 h or 24 h of PEG.

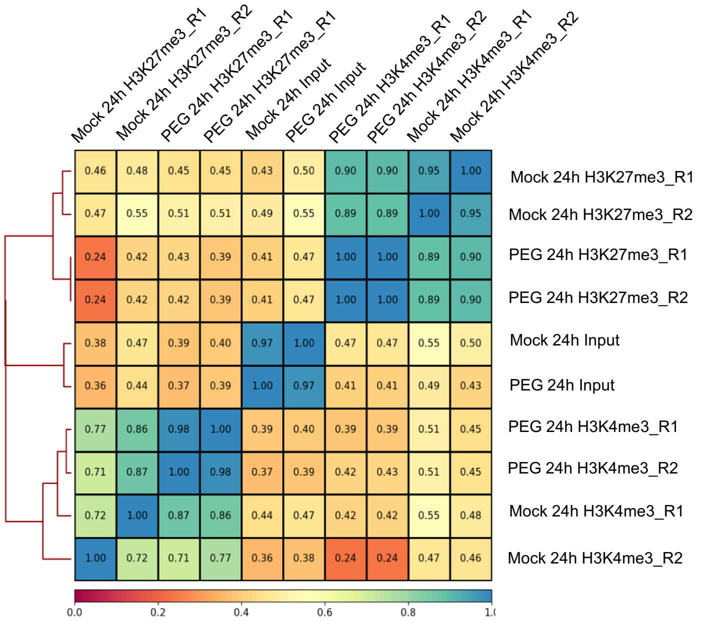

Figure S8: Hierarchical clustered correlation matrix for Mock and PEG-treated replicates. Values are based on ChIP-seq profiling data for H3K4me3 and H3K27me3 histone modifications.

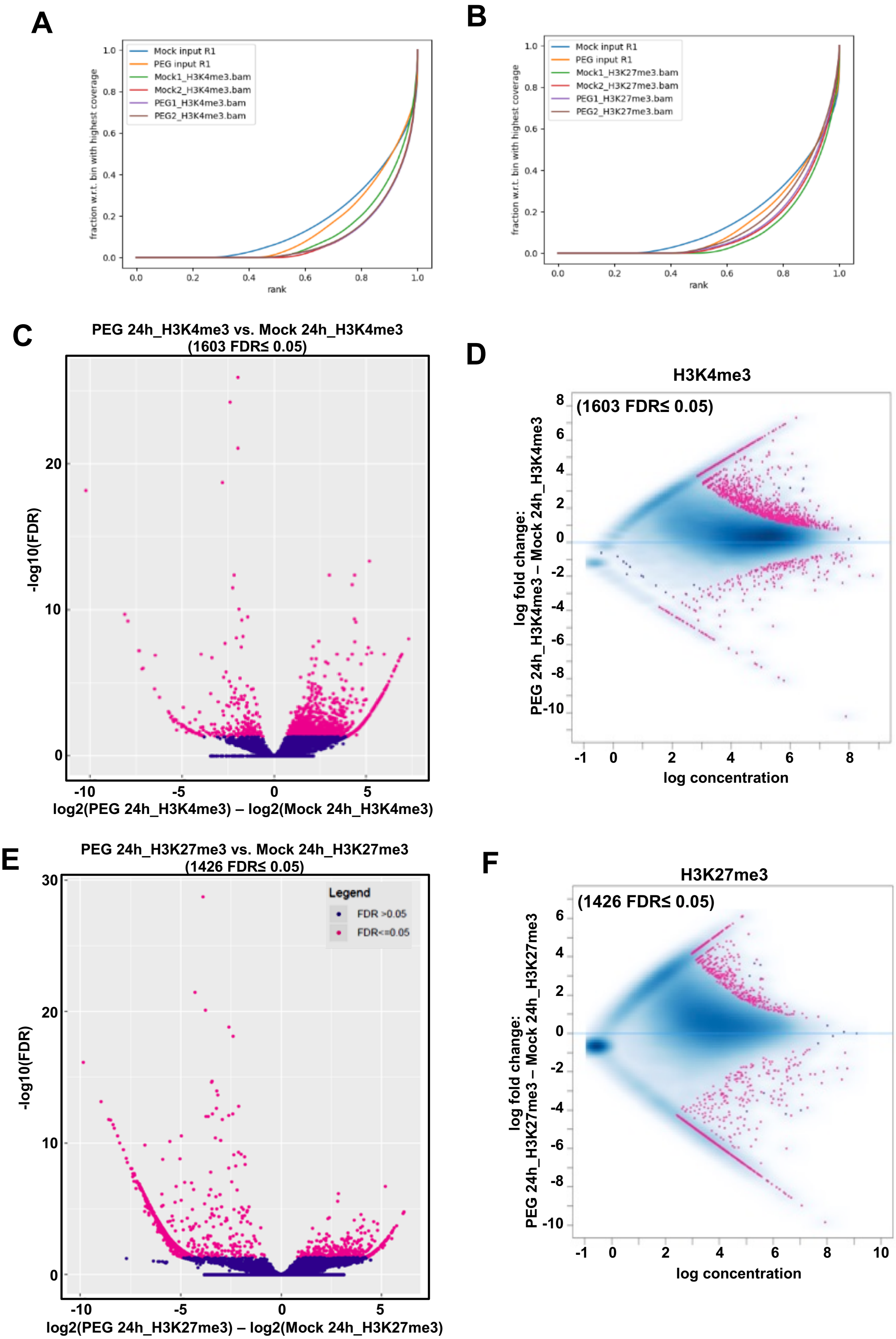

Figure S9: ChIP-seq analysis of rapeseed in response to PEG treatment. **A, B)** Fingerprint profiles of H3K4me3 (A) and H3K27me3 (B) for Mock and PEG-treated datasets, illustrating distinct chromatin binding patterns for each condition. **C**) Volcano plot for differential binding of H3K4me3 in Mock and PEG-treated ChIP-Seq samples for 24 hours. **D**) MA plots for H3K4me3 binding regions [p adj <0.05, fold change (fc) > 1]. **E)** Volcano plot for differential binding of H3K27me3 in Mock and PEG-treated ChIP-Seq samples for 24 hours. **F)** MA plots for H3K27me3 binding regions [p adj <0.05, fold change (fc) > 1].

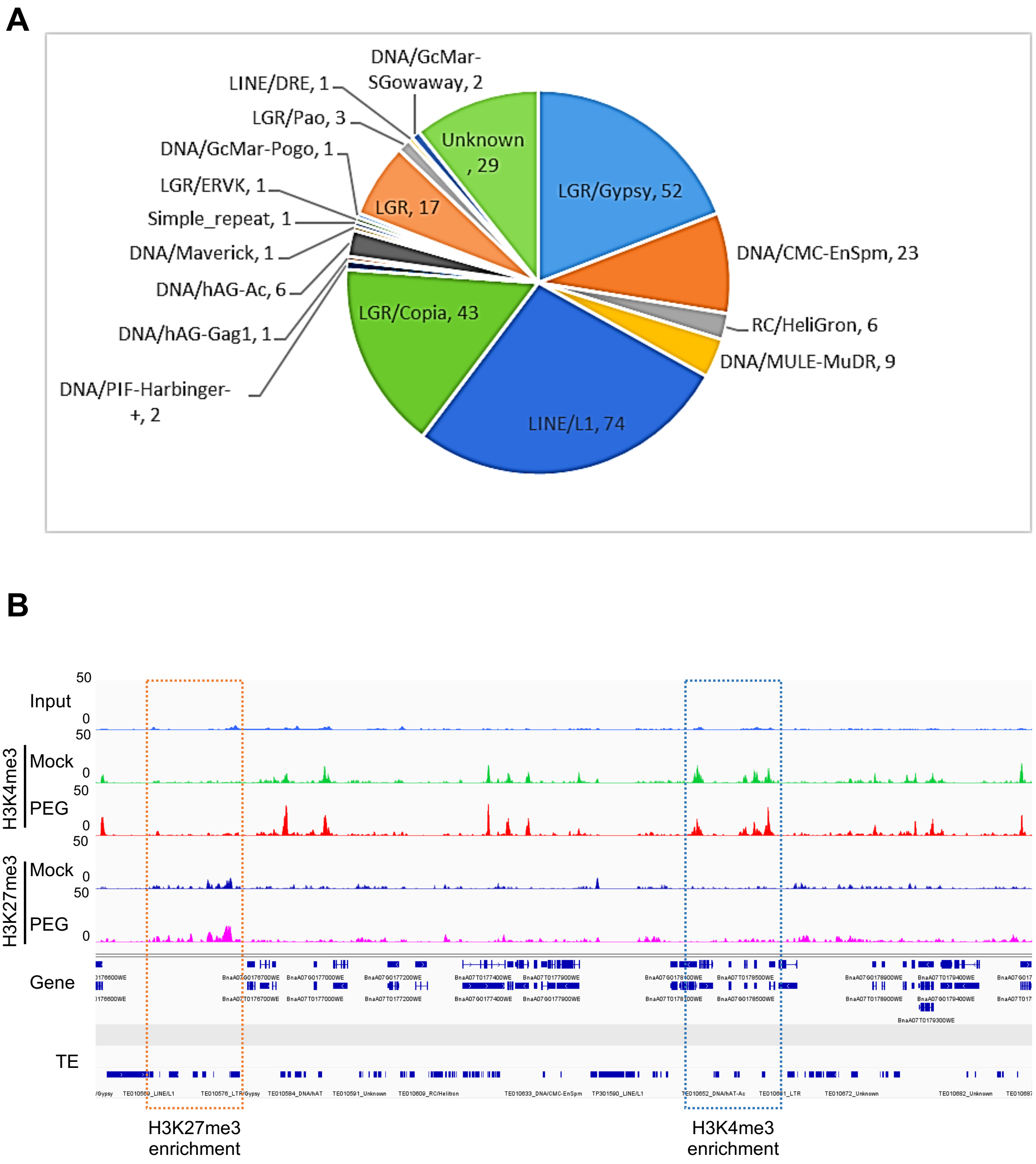

Figure S10. Transposons showing differential enrichment of H3K4me3 marks. A) Transposon categories with differential enrichment of H3K4me3 marks in response to PEG treatment. **B)** IGV (Integrative Genomics Viewer) image displaying the levels of H3K4me3 and H3K27me3 on TEs in the randomly selected rapeseed genome.

A

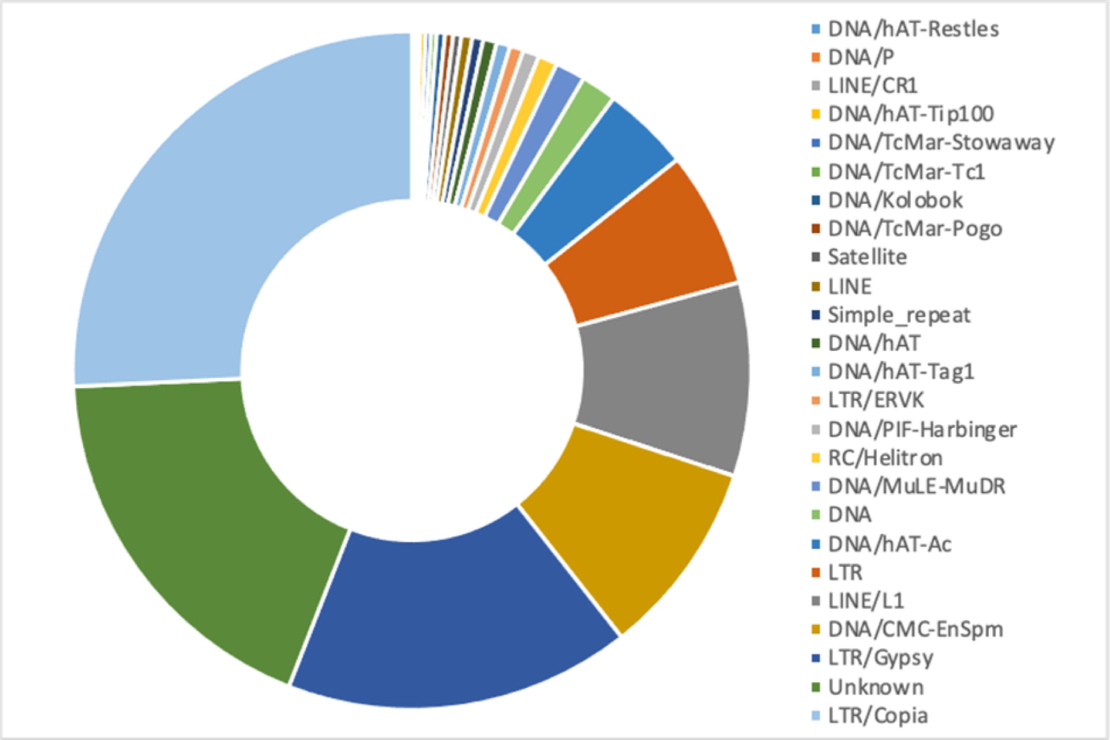

B

**Class**	**Group**	**Numbers**
**Retrotransposon**	LTR	50
	LTR/ERVK	5
	LTR/Copia	198
	LTR/Gypsy	127
	LINE	4
	LINE/CR1	1
**DNA Transposons**	DNA/hAT-Restles	1
	DNA/P	1
	DNA/hAT-Tip100	2
	DNA/TcMar-Stowaway	2
	DNA/TcMar-Tc1	2
	DNA/Kolobok	3
	DNA/TcMar-Pogo	3
	DNA/hAT	5
	DNA/hAT-Tag1	5
	DNA/PIF-Harbinger	6
	RC/Helitron	7
	DNA/MuLE-MuDR	11
	DNA	13
	DNA/hAT-Ac	32
	DNA/CMC-EnSpm	72
	Satellite	3
	Simple repeat	4
**Unknown Transposons**		141
**Total**		**768**

Figure S11. Transposons showing differential enrichment of H3K27me3 marks in response to PEG treatment. A) Transposon categories with differential enrichment of H3K27me3 marks in response to PEG treatment. **B)** Groups of transposons with differential H3K27me3 enrichments.

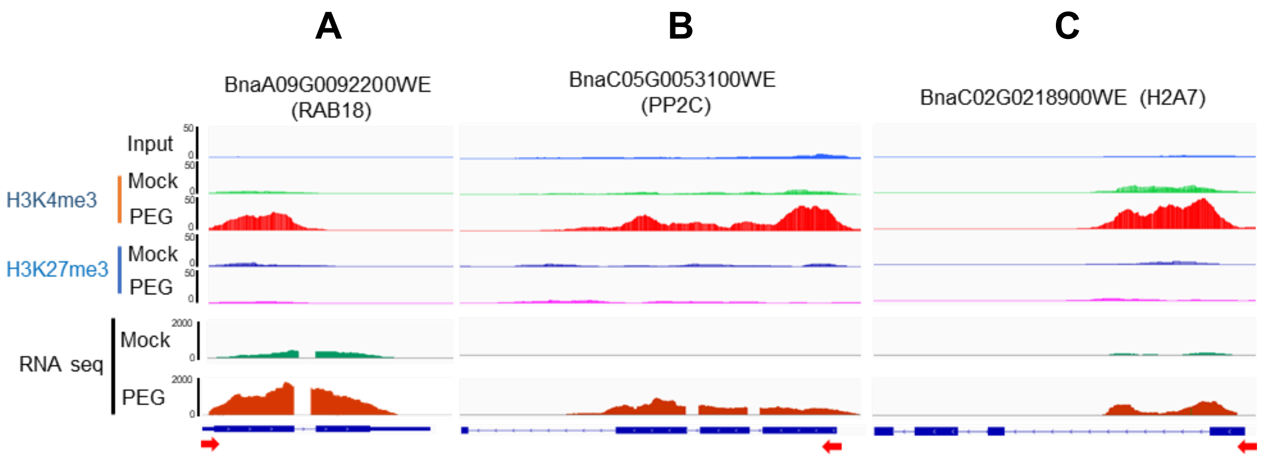

Figure S12: ChIP-Seq and RNA-seq tracks of H3K4me3 and H3K27me3 enrichments in three selected stress-induced rapeseed genes. A) *BnRAB18*, **B)** *BnPP2C*, **C)** *BnH2A7*.

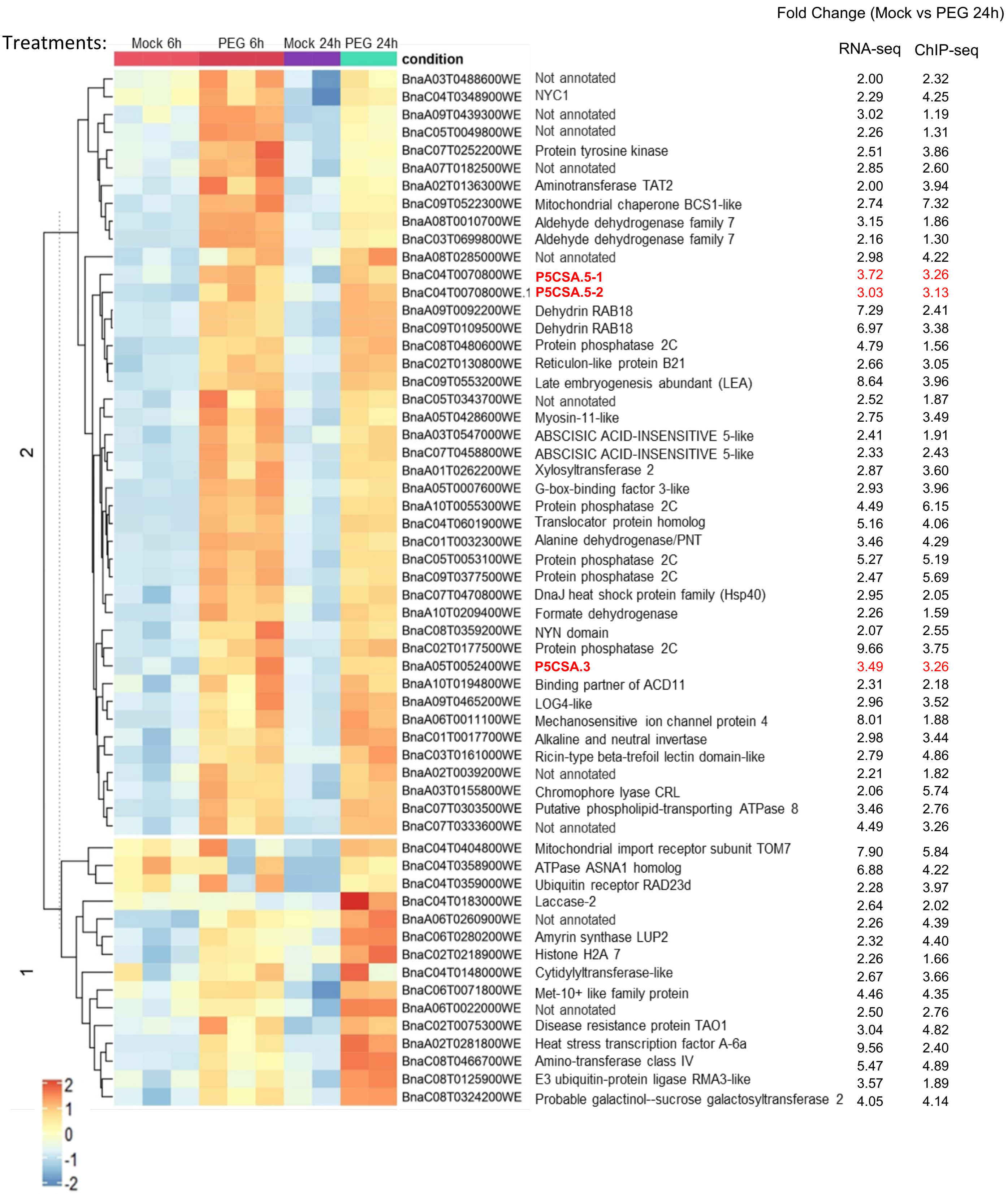

Figure S13: Heatmap of the RNA-seq transcript levels and ChIP-seq changes of the 58 PEG-induced genes that showed H3K4me3 enrichment in response to 24h PEG treatment.

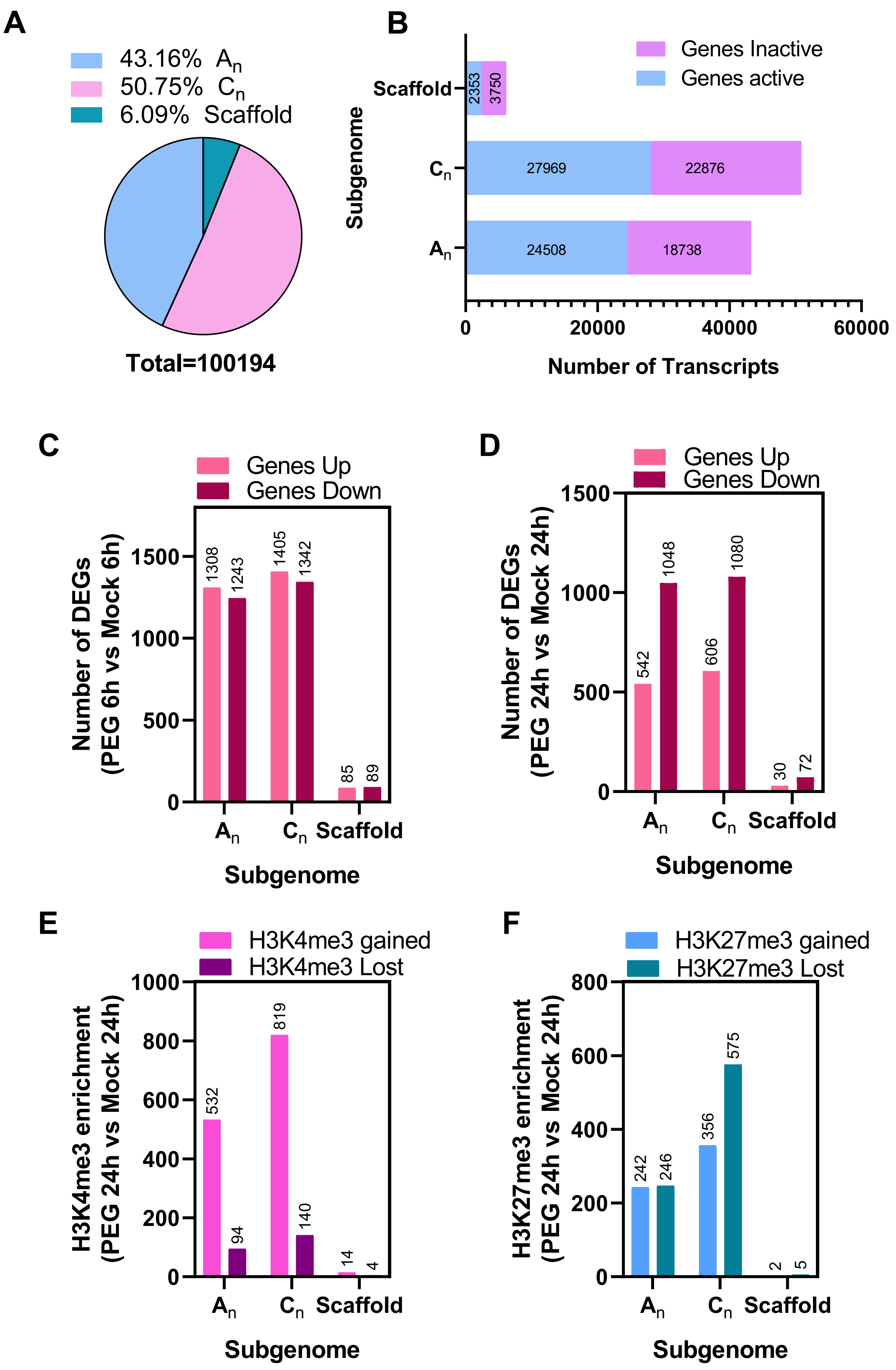

Figure S14: Asymmetrical distribution of epigenomic marks in the An and Cn subgenomes of *Brassica napus*. A) Distribution of An, Cn subgenomes ands scaffold region. **B)** Expressed genes in the subgenomes. **C,D)** Differentially expressed genes in the An and Cn subgenomes after 6 h (C) and 24 h (D) of PEG treatment. **E,F)** Enrichment of H3K4me3 (E) and H3K27me3 (F) marks in the An and Cn subgenomes after 24 h of PEG treatment.

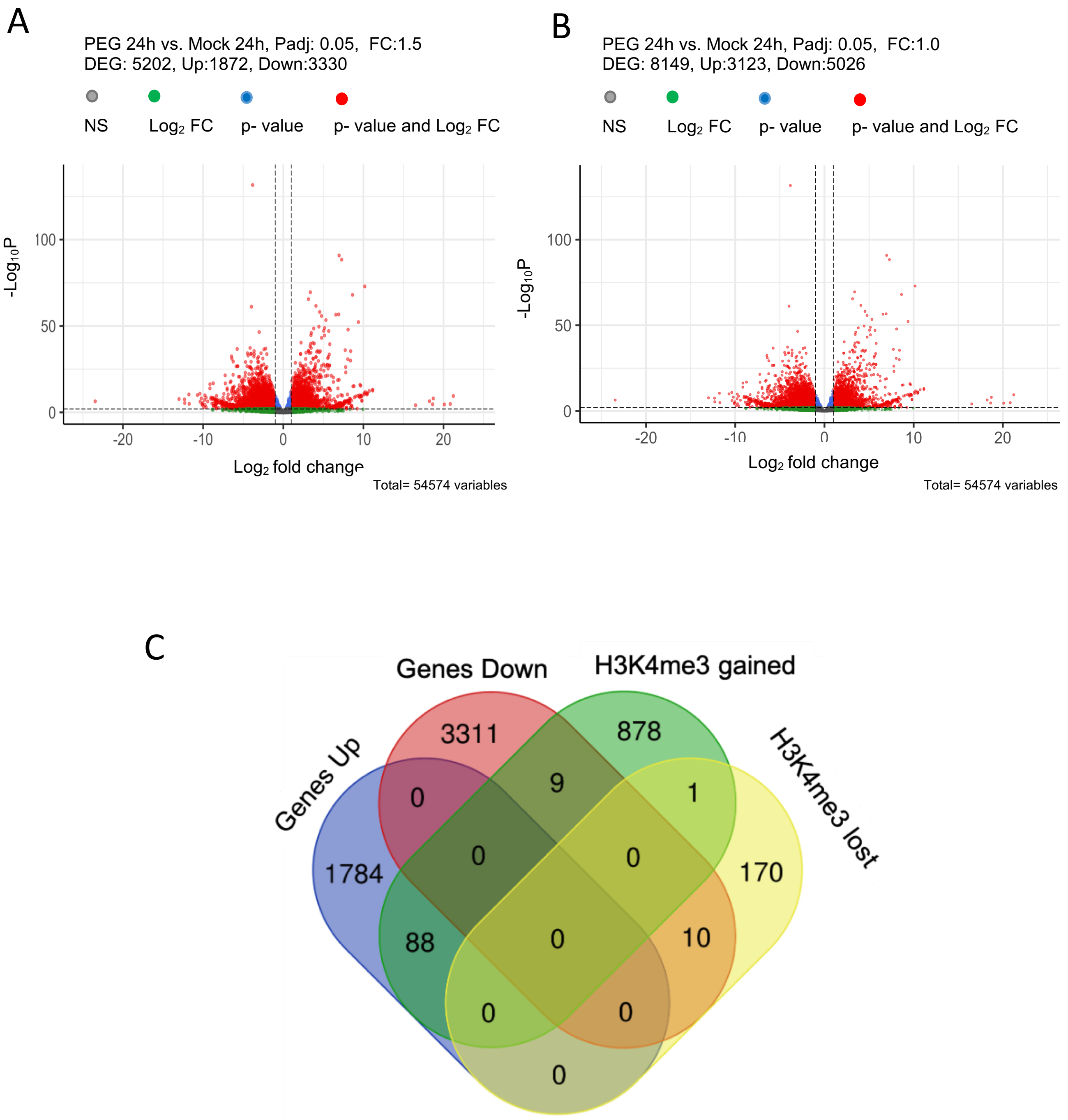

Figure S15: Integration of Rapeseed ChIP-Seq and RNA-Seq Data with reduced stringency. A,B) Volcano plot Analysis of differentially expressed genes (DEGs) in Mock and PEG-treated samples after 24h of PEG treatment. A) FC:1.5, Padj:0.05., B) FC:1.0, Padj:0.05.). The list of genes is provided in Dataset 14. **C)** Venn diagram illustrating the overlap between H3K4me3 targets and DEGs in response to 24 hours of PEG treatment with FC 1.5. Note that The analysis identified 88 genes showing upregulation accompanied by H3K4me3 gain, 10 genes with downregulation and H3K4me3 loss, and 9 genes with downregulation but H3K4me3 gain. The complete list and detailed descriptions are provided in Dataset_15.

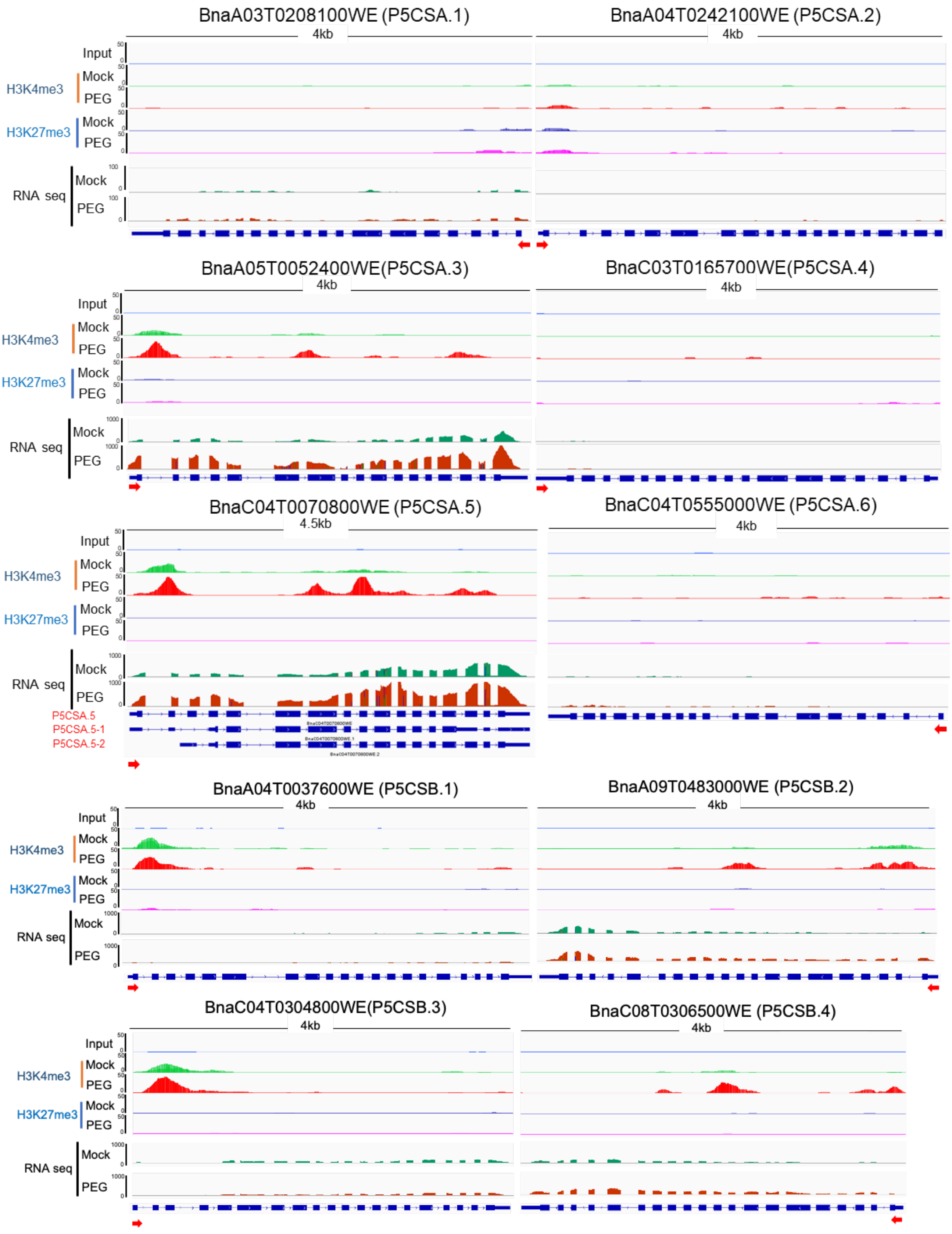

Figure S16: H3K4me3 and H3K27me3 enrichments of *BnP5CSA* and *BnP5CSB* genes in rapeseed. The Y-axis represents normalized read counts of H3K4me3 and H3K27me3 and RNAseq reads within the locus (X-axis), with the direction of transcription indicated by arrowheads .
